# Master Equation Studies of the Unimolecular Decay of Thermalized Methacrolein Oxide: The Impact of Atmospheric Conditions

**DOI:** 10.1021/acs.jpca.3c00542

**Published:** 2023-05-10

**Authors:** Hyun Kyung Lee, Pitchaya Chantanapongvanij, Rory R. Schmidt, Thomas A. Stephenson

**Affiliations:** Department of Chemistry and Biochemistry, Swarthmore College, 500 College Avenue, Swarthmore, Pennsylvania 19081, United States

## Abstract

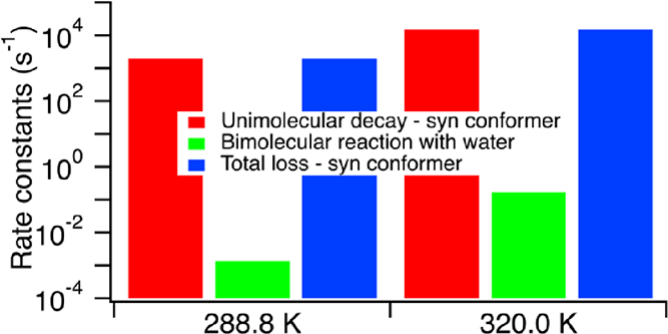

Master equation simulations of the unimolecular reaction dynamics of the Criegee intermediate methacrolein oxide (MACR oxide) have been performed under a variety of temperature and pressure conditions. These simulations provide insight into how the unimolecular kinetics vary across temperatures spanning the range 288–320 K. This work has incorporated a new potential energy surface and includes the anti-to-syn and cis-to-trans conformational dynamics of MACR oxide, as well as the unimolecular reactions to form dioxirane and dioxole species. The competition between the unimolecular reactivity of MACR oxide and previously documented bimolecular reactivity of MACR oxide with water vapor is explored, focusing on how this competition is affected by changes in atmospheric conditions. The impact on the role of MACR oxide as an atmospheric oxidant of SO_2_ is noted.

## Introduction

Hydrocarbons are emitted into the atmosphere in large quantities, from both biogenic and anthropogenic sources. Methane is the most abundant of such species, followed by the unsaturated hydrocarbon isoprene (2-methyl-1,3-butadiene). Biogenic emissions of isoprene are estimated to exceed 500 Tg per year,^1^ while the total emissions from anthropogenic sources (generally from vehicular exhaust and exhaled breath from humans) are much smaller, ≈100 Tg per year.^2^

**Scheme 1:**
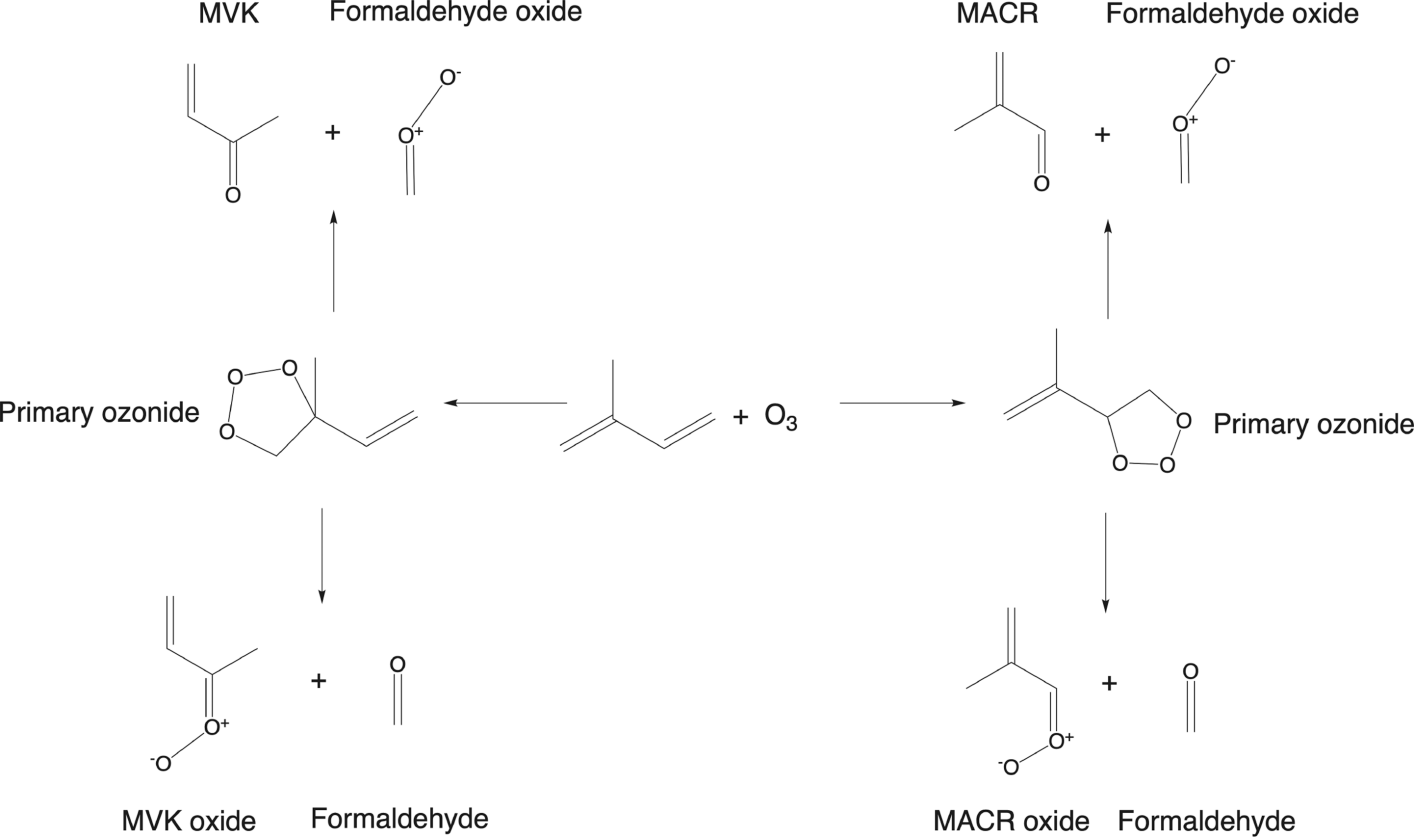
Ozonolysis of Isoprene, Showing the Formation of Two Possible Primary Ozonides and the Subsequent Decomposition to Produce Formaldehyde, Methyl Vinyl Ketone (MVK), Methacrolein (MACR), and the Criegee Intermediates: Formaldehyde Oxide, MVK Oxide, and MACR Oxide

As a class, alkenes are subject to numerous reactions in the atmosphere that can lead to their removal and to the formation of other reactive species. These include reactions with ozone (O_3_), hydroxyl radical (OH), and nitrate radicals (NO_3_).^3,4^ Reaction with ozone—ozonolysis—is a critical reaction in the atmosphere since it depletes an important oxidant and, in some cases, can lead to the formation of another, the hydroxyl radical, OH. Ozonolysis proceeds through the formation of a primary ozonide, in which O_3_ adds across the double bond.^5^ Primary ozonides are formed with a substantial amount of internal energy, which results in rapid, exothermic decomposition to form a carbonyl compound and, as a coproduct, a carbonyl oxide compound, historically known as a Criegee intermediate.^5^ In Scheme 1, we show this reaction sequence for the case of the ozonolysis of isoprene.

Since isoprene contains two carbon–carbon double bonds, there are two possible sites for the formation of a primary ozonide, and each site can lead to the formation of unique decomposition products, as illustrated in Scheme 1. Three possible Criegee intermediates are formed: formaldehyde oxide, methyl vinyl ketone (MVK) oxide, and methacrolein (MACR) oxide. These products are paired with the required carbonyl coproducts: methyl vinyl ketone, methacrolein, and formaldehyde.^6,7^ The Criegee intermediates are formed with significant amounts of excess internal energy and, under atmospheric conditions, will meet one of several fates: stabilization due to collision with inert atmospheric gases (e.g., N_2_, which carries away excess energy), unimolecular decay (which sometimes results in the formation of hydroxyl radical, OH),^8−12^ bimolecular reaction with trace atmospheric gases such as water vapor or SO_2_,^13−27^ or some combination of all three.

Significant advances have been made in the past decade in the understanding of the unimolecular reactivity of stabilized Criegee intermediates, based on detailed experimental and theoretical studies. Among the important kinetic pathways followed by Criegee intermediates under thermal conditions are: conformational dynamics (involving rotation about one or more carbon–carbon or carbon–oxygen bond within the molecule), hydrogen transfer reactions (which can lead to the formation of OH radicals via hydroperoxide formation), ring-closing reactions (leading to the formation of dioxirane structures), and alternative ring-closing mechanisms (leading to formation of dioxole structures).^28,29^ Many of the dioxole and dioxirane structures are believed to undergo further unimolecular decay. With respect to the ozonolysis of isoprene, the majority of the experimental and theoretical studies have focused on the properties and reactivity of MVK^6,25−27,30−40^ and formaldehyde oxides,^14,41−52^ with MACR oxide being relatively less studied.^24,32−35,53−57^ At the same time, it is clear that MACR oxide can play an important role in the ozone-derived chemistry of isoprene: as described by others, and discussed below, anti-MACR oxide is unusually long-lived (τ ≈ 0.10 s at 298 K) with respect to unimolecular decay,^24,29,53^ making this Criegee intermediate a potentially potent contributor to bimolecular chemistry in the atmosphere.

**Figure 1:**
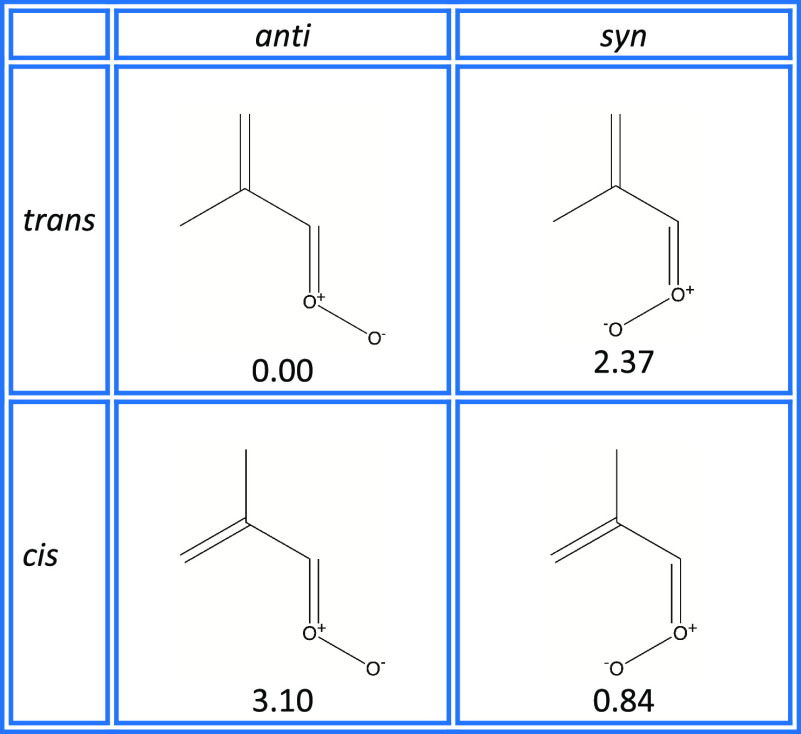
Conformers of methacrolein oxide. The relative stabilities of the conformers are shown (kcal/mol), calculated at the CCSD(T)/cc-pVQZ//B2PLYP-D3/cc-pVTZ level of theory.

Methacrolein oxide exists as four conformers, distinguished by rotation about the central carbon–carbon bond (conventionally labeled as cis- and trans-conformers), and by rotation about the carbon–oxygen bond (conventionally labeled as syn- and anti-conformers).^53^ These four structures are shown in Figure 1. In Table 1, the relative energies of these conformers are presented—from both literature sources, and the values that we have derived from our computational methods (described below). Kuwata and Valin,^55^ building on initial studies by Zhang et al.,^58,59^ described the conformational dynamics and performed initial master equation modeling that determined unimolecular decay products and branching ratios resulting from the decay of MACR oxide. Unlike MVK oxide,^29,30^ none of the conformers of MACR oxide are subject to the efficient 1,4-hydrogen shift reaction that limits the lifetime of some MVK oxide conformers under atmospheric conditions. The unimolecular lifetime of MACR oxide is determined by a 1,5-ring-closing reaction to form a dioxole structure (syn-conformer only) and a 1,3-ring-closing reaction to form a dioxirane structure (syn- and anti-conformers).^29,53,55^ As examined previously, and discussed in more detail below, these ring-closing mechanisms have kinetic constants that differ by orders of magnitude, generating conformer specific unimolecular decay rates. The syn- to anti-conversion transition states are sufficiently high energy (>20 kcal/mol) that the conversion between these conformers is negligible under thermal conditions.

**Table 1:** Relative Energies of Methacrolein Oxide Conformers

	relative energies (ZPE corrected), kcal/mol				
anti-trans	0.00^a^	0.00^b^	0.0^c^	0.0^d^	0.0^e^
anti-cis	3.10	3.18	3.2	3.2	3.0
syn-trans	2.37	2.50	2.7	2.0	2.8
syn-cis	0.84	0.91	1.1	0.5	1.5

a CCSD(T)/cc-pVQZ//B2PLYP-D3/cc-pVTZ, this work.

b CCSD(T)-F12/CBS(TZ-F12,QZ-F12)//B2PLYP-D3/cc-pVQZ, ref (53).

c CBS-QB3, ref (55).

d B3LYP/6-31G(d,p), ref (58).

e CCSD(T)/6-31G(d)+CF, ref (58).

Vansco et al. synthesized MACR oxide in the laboratory for the first time and recorded an ultraviolet electronic absorption spectrum that consisted of overlapping contributions from all four conformers.^53^ These investigators also refined the structures and energies of the conformers using a high level of electronic structure theory (see Table 1). Rapid formation of dioxole structures resulting from the unimolecular decay of MACR oxide was detected in a separate study by Vansco et al., verifying the presumed dominant atmospheric sink for the syn-conformers of MACR oxide.^33^ On the other hand, the anti-conformers of MACR oxide are found to be long lived (*k*_uni_ ≈ 10 s^–1^ at 298 K), since the dominant unimolecular decay mechanism is the relatively slow formation of a dioxirane species. As a result, bimolecular reactivity of anti-MACR oxide is competitive with unimolecular decay and is found to have a potential impact on the atmospheric oxidation of SO_2_. Oxidation of SO_2_ by stabilized Criegee intermediates is estimated to account for as much as 40% of the H_2_SO_4_ created in the nighttime atmosphere, as well as up to 10% of daytime H_2_SO_4_ formation.^60^

Analysis of field studies examining sulfate aerosol formation in power plant plumes has identified the Criegee intermediates derived from the ozonolysis of isoprene (formaldehyde oxide, MVK oxide, and MACR oxide) as important contributors to the chemistry of this complex environment.^61^ Specifically, models of secondary sulfate aerosol formation are sensitive to the assumed degree of competition between bimolecular reactivity of isoprene-derived Criegee intermediates with water vapor and SO_2_ and the unimolecular decay of the Criegee intermediates. This competition appears in stark terms in the recent experimental work of Lin et al.,^24^ in which the reaction of anti-MACR oxide with water vapor is found to be slower by almost two orders of magnitude than previous theoretical estimates.^62^ The reaction with water vapor is the dominant loss mechanism for anti-MACR oxide at 298 K for a range of humidity conditions.^24^ On the other hand, for syn-MACR oxide, unimolecular decay dominates all other loss mechanisms.

The central concern of our work is to examine in more detail the conformer specific unimolecular kinetics of MACR oxide under atmospheric conditions and to compare the rates of unimolecular decay with those of bimolecular reaction with water and water dimer. We are specifically focused on determining whether variations in atmospheric conditions dictate a significant change in the competition between unimolecular and bimolecular chemistry. Since biogenic isoprene emissions approximately double with every 7-degree Kelvin increase in ambient temperature,^63^ it is critical to understand the temperature dependence of the decay of the isoprene ozonolysis products. In this work, we illustrate the variations in kinetics that occur when operating under realistic atmospheric conditions (i.e., other than 1013 mbar pressure, 298 K).

## II Computational Details

All of our quantum chemistry calculations used Gaussian 16 to determine the energies, structures, and harmonic vibrational frequencies of the minima and transition-state species on the MACR oxide potential energy surface.^64^ Initial structures were found using the B3LYP functional and cc-PVTZ basis set and were refined using the B2LYPD3 functional and cc-PVTZ basis set. The B2PLYP functional in particular has been found to be a good predictor of structures and vibrational frequencies for modest sized hydrocarbon species in a number of benchmark studies, once implemented with dispersion corrections.^65,66^ Single-point energies, based on B2PLYPD3/cc-PVTZ geometries, were determined using the CCSD(T) method and a somewhat larger basis set, cc-PVQZ. In Table 1, we present a comparison of a series of studies of the relative energies of the MACR oxide conformers. The mean deviation of the energies found in this work is <0.1 kcal/mole from that of Vansco et al.,^53^ with the latter being a calculation with a more robust basis set. In the Supporting Information, we provide the Cartesian coordinates for the optimized structures used in these calculations.

We used MESMER 6.0 to solve the master equation for the isomerization of the conformers of thermalized MACR oxide and the unimolecular decay of each of the conformers.^67^ Our input into the MESMER calculations was the B2PLYPD3 structures and vibrational frequencies, and the CCSD(T) single-point energies. The exponential down model is used to treat the collisional stabilization/activation process by N_2_ gas. The average energy transferred per collision was assumed to be 200 cm^–1^. For N_2_, the Lennard-Jones parameters were σ = 3.74 Å and ε = 82 K. For all hydrocarbon species, we assume σ = 6.29 Å and ε = 358.0 K.^55^ Tunneling is included in the MESMER calculations using a one-dimensional Eckart model. All species are assumed to be thermalized with a Boltzmann distribution of energies characterized by the ambient temperature.

We find (see Results and Discussion, below) that the thermal rates extracted from the MESMER simulations are sensitive to the treatment of the hindered rotation about methyl groups and the torsional motion about the central carbon–carbon bond in MACR oxide. In our B2PLYPD3 frequency calculations, we calculate harmonic frequencies corresponding to both of these motions. Since our RRKM theory-based kinetic simulations should be strongly affected by the density of states in both stable species and transition states, we have also calculated one-dimensional hindered rotor potentials for the methyl rotors in MACR oxide, the transition-state structures, and the dioxirane and dioxole product molecules. We also determined the intramolecular potentials associated with the carbon–carbon bond torsions in all the relevant species. These hindered rotation and bond torsion potentials are used in our MESMER simulations to calculate the densities of states in lieu of the harmonic vibrational frequencies corresponding to these low-frequency motions. Plots of the hindered rotor potentials calculated in our work and used in the master equation simulations are included in the Supporting Information.

## III Results and Discussion

As shown in Figure 1, MACR oxide has four conformers, distinguished by rotations about the carbon–oxygen bond (syn- and anti-conformers) and the central carbon–carbon bond (cis- and trans-conformers). These four structures have ground-state energies that lie within 3.2 kcal/mole of one another when calculated at the CCSD(T)/cc-pVQZ level of theory (Table 1). Note that the anti-trans conformer is the most stable, while the anti-cis is the least stable.

**Scheme 2:**
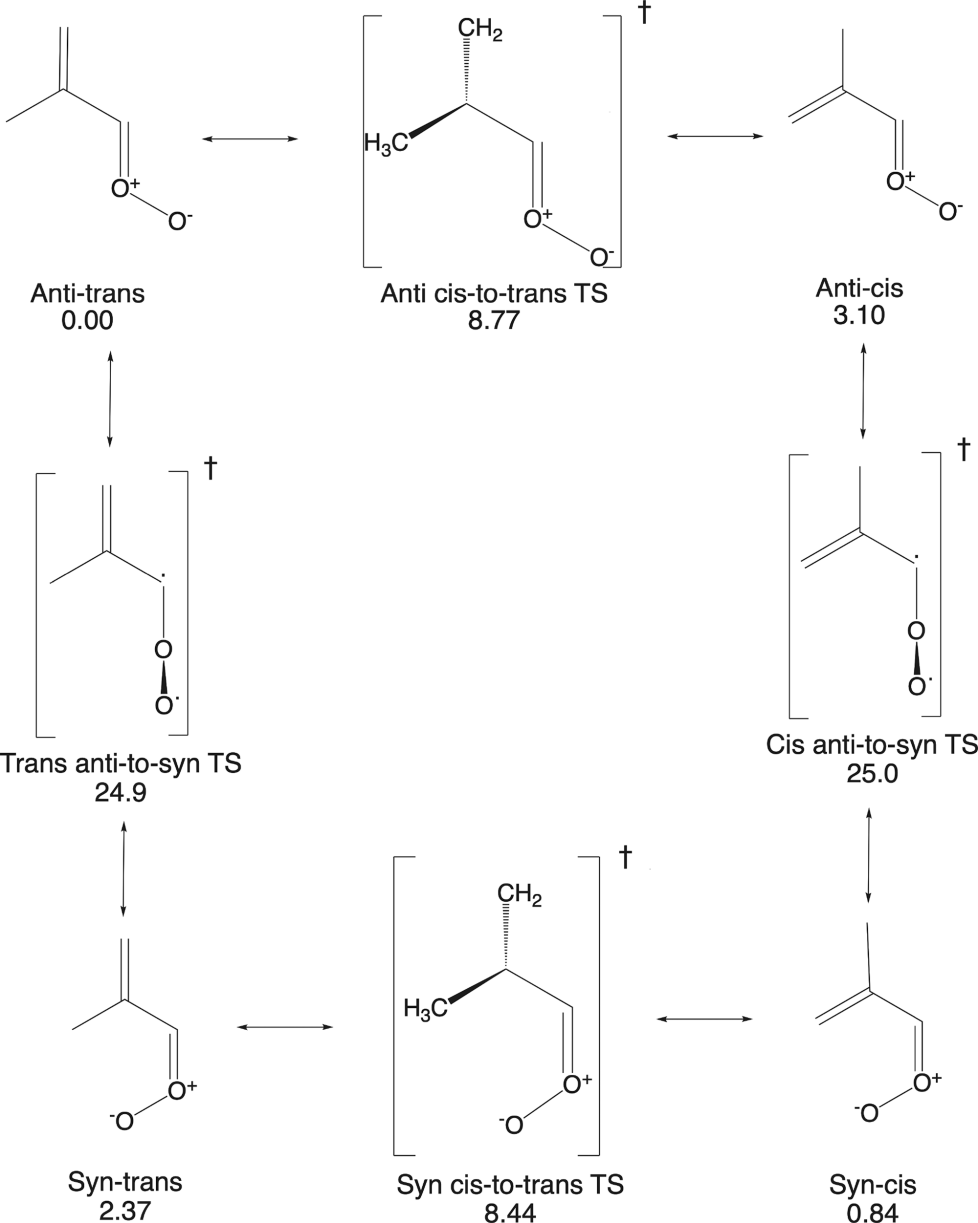
Methacrolein Oxide Isomerization Pathways and Transition States Energies (ZPE corrected and relative to the anti-trans conformer) are shown in kcal/mol.

Scheme 2 shows the barriers to conversion between the conformers of MACR oxide. The cis- and trans-conformers have the modest barriers to isomerization (<9 kcal/mole), while the barriers to conversion between the syn- and anti-conformers are much larger (>20 kcal/mole). The high barriers that separate the syn- and anti-conformers prevent interconversion of these species on timescales that are competitive with the other unimolecular processes (discussed below) important in MACR oxide. For example, we find that, in the high-pressure limit at 298.0 K, the largest unimolecular rate constant for an anti-to-syn (or syn-to-anti) conversion is for the conformational change anti-cis → syn-cis MACR oxide. For this process, *k*_uni_ = 1.48 × 10^–3^ s^–1^, corresponding to a lifetime of 674 s. This conformational change is more than 250× slower than the next slowest unimolecular reaction involving MACR oxide. We find no evidence of population transfer between anti- and syn-conformers in our kinetic simulations. In our analysis, we will consider the syn- and anti-conformers to be effectively separate populations.

**Figure 2:**
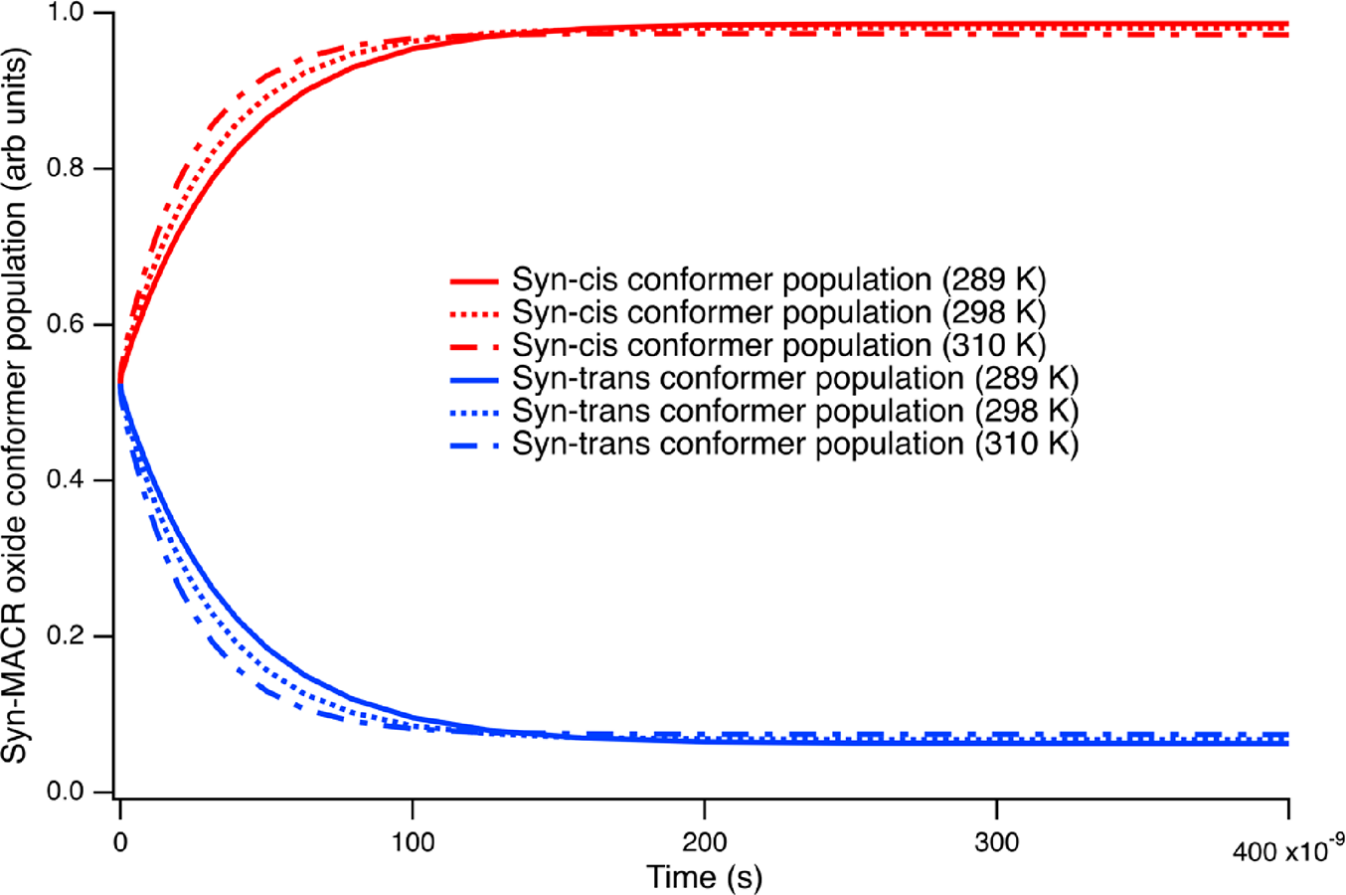
Initial population evolution of syn-cis (red) and syn-trans (blue) conformers at temperatures of 288.8 K (solid lines), 298 K (dashed lines), and 310 K (dot-dash lines). The buffer gas is N_2_ at a pressure of 1013 mbar in all cases.

On the other hand, as illustrated on Figure 2, the cis- and trans-conformers of MACR-oxide are in rapid equilibration. Under the average conditions appropriate for atmospheric chemistry at the surface of the Earth (1.013 bar pressure, and an average temperature of 288.8 K),^68^ the syn-cis and syn-trans conformers are found to reach a transient equilibrium with a rate constant of 2.6 × 10^7^ s^–1^. This equilibrium favors the lower energy conformer, syn-cis, with a transient population ratio of 15.8. Similar results are observed for the anti-cis and anti-trans conformers (see the Supporting Information); although in this case, the rate of equilibration is faster (the cis-to-trans barrier is <6 kcal/mol), and the discrimination between conformers is more dramatic (the energy difference between the anti-conformers is larger). As seen in Figure 2 and Figure S1, these processes are dependent on atmospheric conditions. At temperatures that are characteristic of warmer climates, 298 and 310 K, the equilibration rate constants for the syn-conformers increase to 3.3 × 10^7^ and 4.3 × 10^7^ s^–1^, respectively, while the population ratios between syn-cis and syn-trans conformers change to 14.4 and 13.0, respectively. In the Supporting Information (Table S1), we present a set of data summarizing population equilibration rates and transient population ratios for the syn- and anti-conformers of MACR oxide at a range of temperatures in the troposphere. Included in these data are results from a simulation carried out at a temperature and pressure (259.3 K, 542 mbar)^68^ characteristic of an altitude of 5 km. This data point is of potential interest to field studies that have detected isoprene-derived products at altitudes above the surface.^61^ In Figure S1, we plot data comparable to Figure 2, but showing the initial equilibration of the anti-MACR conformers at 288.8, 298, and 310 K and *P* = 1013 mbar.

**Figure 3:**
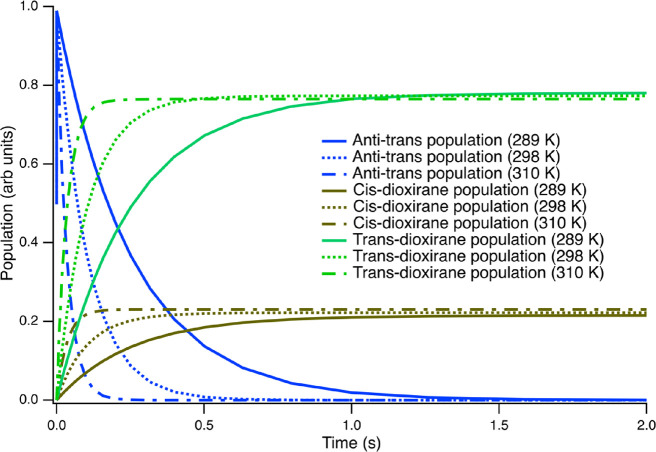
Decay of anti-trans methacrolein oxide (blue traces) to form trans-dioxirane (green traces) and cis-dioxirane (dark green traces) at temperatures of 288.8 K (solid lines), 298 K (dashed lines), and 310 K (dot-dash lines). The buffer gas is N_2_ at a pressure of 1013 mbar in all cases.

**Scheme 3:**
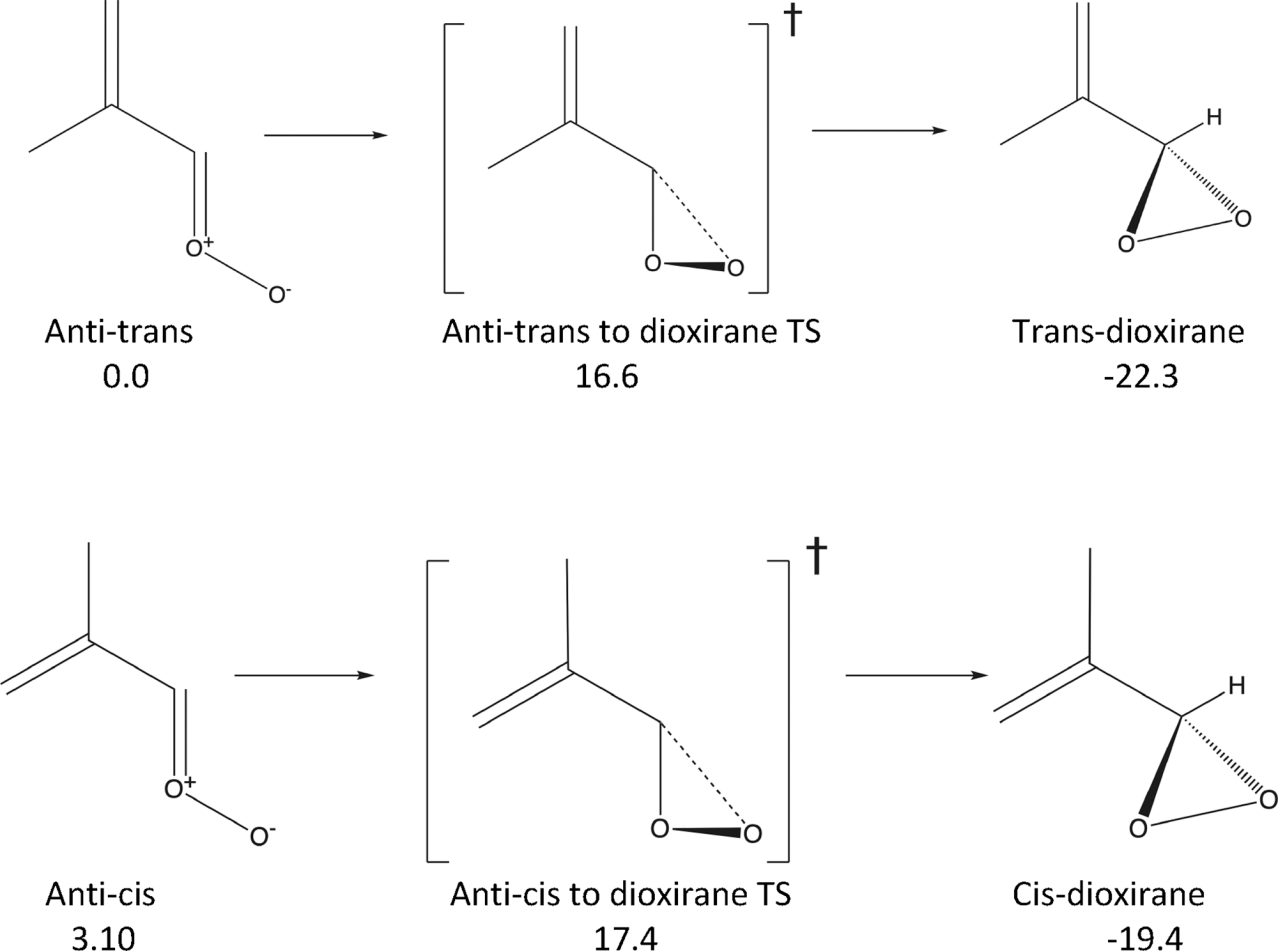
Unimolecular Decay Pathways Followed by Anti-Trans and Anti-Cis Methacrolein Oxide Energies (ZPE corrected and relative to the anti-trans conformer) are shown in kcal/mol.

We turn now to the unimolecular decay of the syn- and anti-populations at times after the equilibration of the cis- and trans-conformers. In Scheme 3, we display the principal unimolecular decay pathway followed by the anti-cis and anti-trans conformers. For these molecules, a 1,3-ring closing mechanism results in the formation of dioxirane structures. As noted in the scheme, these pathways occur over transition states with energies of ≈15 kcal/mol. The magnitude of these barriers compared to the cis-trans isomerization barrier (≈8.5 kcal/mole; see Scheme 2) means that there is a clean separation of timescales between the equilibration of the cis and trans isomer populations and the formation of the dioxirane. In Figure 3, the decay of anti-trans-MACR oxide to form trans-dioxirane is shown at temperatures of 288.8, 298, and 310 K. (Note that because of the rapid pre-equilibration of the populations of the cis- and trans-conformers of MACR oxide, the decay of anti-cis-MACR oxide mimics these curves exactly.) At the average surface temperature, 288.8 K, the anti-conformers undergo unimolecular decay with a rate constant of 3.9 s^–1^. This rate constant increases to 9.7 and 29 s^–1^ at 298 and 310 K, respectively. Anti-conformers form exclusively trans- and cis-dioxirane structures. Both dioxirane structures are accessible and are formed with first-order kinetics, as seen in Figure 3. Due to the lower transition-state energy, the trans-dioxirane structure is the favored product; the equilibrium product ratio is 3.63 at 288.8 K. As the ambient temperature increases, the discrimination in favor of the trans-dioxirane conformer decreases; the trans:cis equilibrium product ratios are 3.49 and 3.32 at 298 and 310 K, respectively. In Table S2, we provide a complete list of the anti-conformer decay rate constants and dioxirane product ratios as a function of atmospheric conditions.

**Figure 4:**
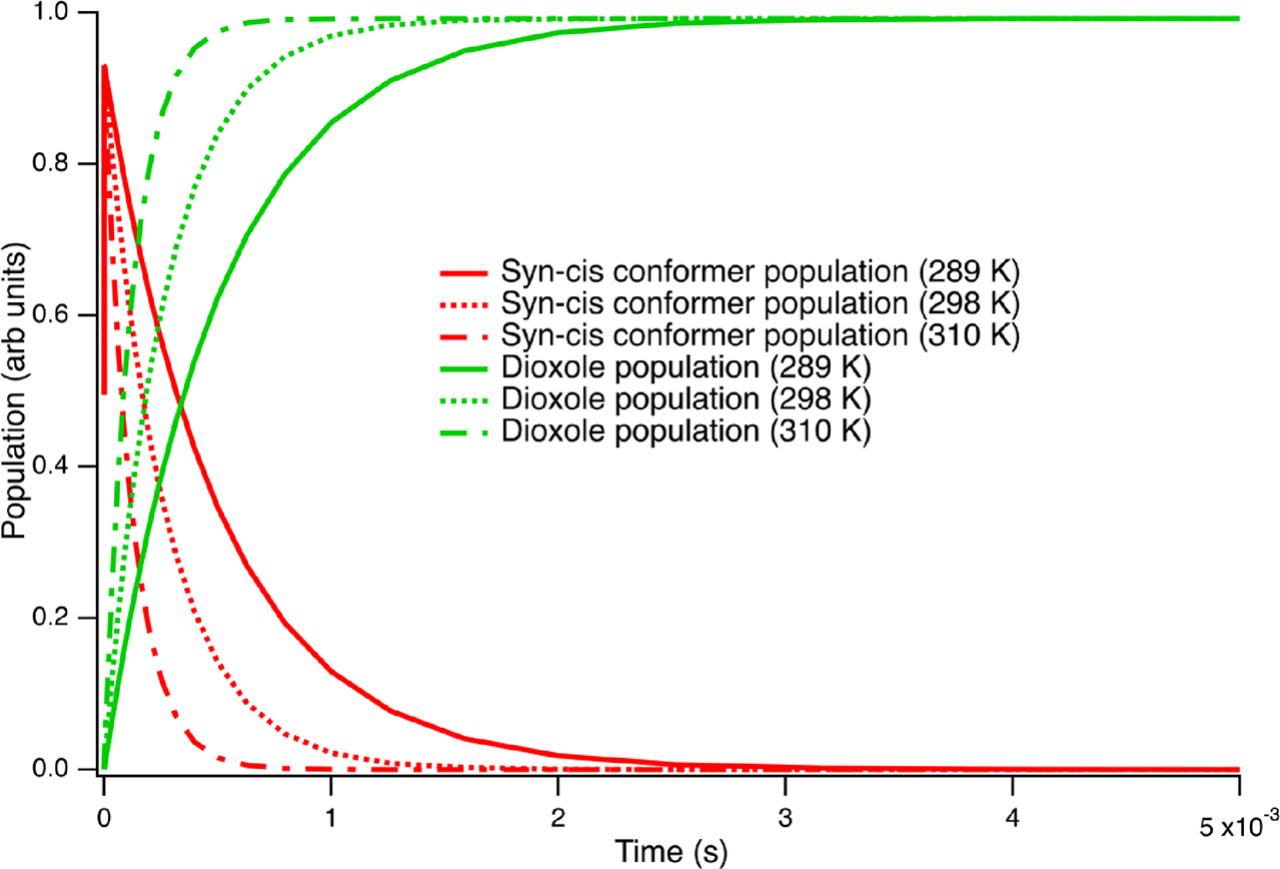
Decay of syn-cis methacrolein oxide (red traces) to form dioxole (green traces) at temperatures of 288.8 K (solid lines), 298 K (dashed lines), and 310 K (dot-dash lines). The buffer gas is N_2_ at a pressure of 1013 mbar in all cases.

**Scheme 4:**
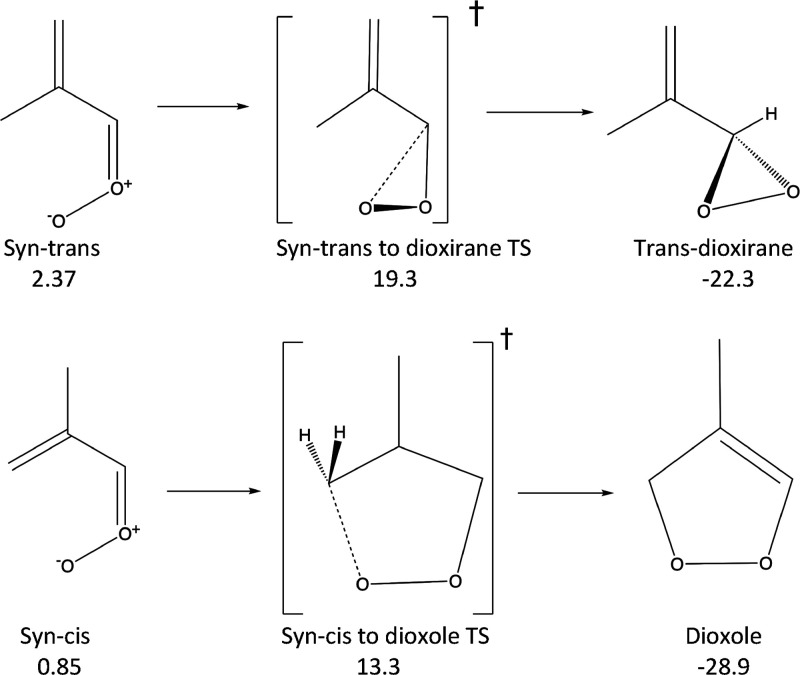
Unimolecular Decay Pathways Followed by Syn-Trans and Syn-Cis Methacrolein Oxide^a^ Energies (ZPE corrected and relative to the anti-trans conformer) are shown in kcal/mol.

In Scheme 4, we show the unimolecular decay pathways available to the syn-conformers. Similar to the anti-conformers (Scheme 3), the syn-trans structure can undergo a 1,3-ring closure mechanism to form the trans-dioxirane structure. Uniquely among the four MACR oxide structures, however, the syn-cis conformers can undergo a 1,5-ring closure to form a dioxole species, with a relatively low transition state energy (Scheme 4). (The syn-cis conformer can also form the cis-dioxirane via a transition state (not shown) with an energy much higher (>25 kcal/mol) than the others considered here.^55^) In Figure 4, the decay of the syn-conformers is shown, which is dominated by the relatively low-energy formation of the dioxole species. At the average surface temperature of 288.8 K, the syn conformers decay with a rate constant of 2.0 × 10^3^ s^–1^, a factor of ≈500× faster than the anti-conformers under the same conditions. As shown in Figure 4, the rate of decay is substantially faster at 298 and 310 K; the first-order rate constants under those conditions are 3.7 × 10^3^ and 8.1 × 10^3^ s^–1^, respectively. In Figure 4, we plot the decay of the syn-cis conformer (red traces). The final population of the dioxole species (green traces) is higher than the initial population of the syn-cis conformer, a result of the complete conversion of sum of the syn-cis and syn-trans populations to the dioxole species. There is no evidence that, under thermal conditions, any of the syn-conformer population decays to form cis- or trans-dioxirane. Rather, the rapid equilibration of the cis- and trans-syn-conformers followed by rapid decay of the syn-cis conformer constitutes effectively 100% of the total unimolecular decay of these conformers. The high-pressure rate constant at 298 K for the formation of dioxirane from the syn-conformers (Table 2) that we extract from the master equation analysis is >10^4^ times smaller than the rate constant to form dioxole. This result is in accord with the lack of any noted transfer of syn-MACR oxide to the dioxirane structure. In Table S2, we provide a complete list of the syn-conformer decay rate constants as a function of atmospheric conditions.

**Table 2:** Unimolecular Rate Constants (s^–1^) in the High-Pressure Limit at 298 K^a^

	hindered rotor potentials^b^	harmonic vibrations^b^	Vereecken et al.^c^	Lin et al.^d^
anti-MACR oxide to dioxirane	9.9	6.1	10.	7.
syn-MACR oxide to dioxirane	0.39	0.14	0.35	
syn-MACR oxide to dioxole	4.0 × 10^3^	2.0 × 10^3^	2.5 × 10^3^	2.6 × 10^3^

a The buffer gas is N_2_ in all cases.

b This work.

c Ref (29).

d Ref (24).

The kinetics of these unimolecular decay pathways have been considered previously, albeit under somewhat different conditions than those presented above. It is useful, however, to benchmark our studies with these previous studies. Vereecken and co-workers reported the unimolecular decay kinetics of a range of Criegee intermediates at 298 K in the high-pressure limit.^29^ In this work, the reactants, products, and transition states were treated within the harmonic oscillator-rigid rotor approximation, with structures optimized at the M06-2X/aug-cc-pVTZ level of theory and single-point energies determined using the CCSD(T)/aug-cc-pVTZ methodology. Similarly, Lin et al., as part of their experimental and theoretical examination of the bimolecular kinetics of MACR oxide with water vapor reexamined the unimolecular decay kinetics.^24^ In this work, structures were optimized with the B3LYP/6-311+G(2d,2p) methodology, followed by single-point energy calculations using the QCISD(T) method with the Dunning basis set extrapolated to the complete basis set (CBS) limit.

To benchmark our potential energy surface for MACR oxide kinetics, and to document the impact of representing rotations of methyl groups and carbon–carbon bond torsions as hindered rotors (rather than harmonic vibrations), we have repeated our MESMER simulations at 298 K and at a series of pressures to extract unimolecular rate constants in the high-pressure limit. The comparative results are shown in Table 2, along with the results of Vereecken et al., and Lin et al. From a comparison of the first two columns of data, we observe that the effect of the additional density of states associated with the hindered rotor potentials on the calculated kinetics can be significant, even when carried out on the same potential energy surface. We believe that the hindered rotor model is a more realistic description of the density of states, and aside from Table 2, all of the data presented here use the former approach. A comparison of the rightmost three columns compares different methodological treatments of the electronic structures and energies, but similar treatments of the densities of states. These data suggest that the barrier to the formation of the dioxole structure is well understood, but that the barrier to the formation of dioxirane is subject to some uncertainty. In their consideration of the unimolecular decay of anti-MACR oxide, Lin et al. estimated that the uncertainties in the barrier height to form the dioxirane product produce potential errors in the high-pressure rate constants for the unimolecular decay as high as a factor of 3,^24^ an estimate consistent with the results shown in Table 2.

To further document the unimolecular reactivity, we have also determined the high-pressure rate constants for the unimolecular decay of MACR oxide at temperatures other than 298 K. In Table 3, we present data demonstrating how the high-pressure rate constants vary with temperature, highlighting especially values pertaining to the average surface temperature (288.8 K)^68^ and somewhat warmer climates (298, 310 K). We also present the rate constants for the unimolecular decay processes at a pressure of 1013 mbar. These data show that the faster MACR oxide reactions are kinetically very close to the high-pressure limit at 1013 mbar. As illustrated in Figure S2 (for 298 K), pressures near 500 mbar are very close to the fall off region for the formation of dioxirane from anti-MACR oxide and the formation of dioxole from syn-MACR oxide. The reactions will move out of the high-pressure limit for pressures <500 mbar. The slow, and kinetically insignificant, reaction of syn-MACR oxide to form dioxirane is not in the high-pressure limit at any pressures of atmospheric relevance.

**Table 3:** Unimolecular Rate Constants (s^–1^) at Different Pressures and Temperatures^a^

	high-pressure limit			1013 mbar		
	288.8 K	298.0 K	310.0 K	288.8 K	298.0 K	310.0 K
anti-MACR oxide to dioxirane	4.0	9.9	30.	3.9	9.7	29.
syn-MACR oxide to dioxirane	0.14	0.39	1.3	8.0 × 10^–2^	0.20	0.62
syn-MACR oxide to dioxole	2.1 × 10^3^	4.0 × 10^3^	8.9 × 10^3^	2.0 × 10^3^	3.9 × 10^3^	8.4 × 10^3^

a The buffer gas is N_2_ in all cases.

To place these results on the unimolecular chemistry in the context of the bimolecular chemistry of MACR oxide with water vapor, we draw on the recent work of Lin et al.,^24^ in which both experiment and theory shed light on this important system. In their work, Lin et al. found that the experimental, effective rate constant for the reaction of water vapor with anti-MACR oxide at 298 K is *k*_water – eff_ = 9 ± 5 × 10^–17^ cm^3^ s^–1^, where *k*_water – eff_ takes into account the impact of reactivity with both water monomers and water dimers,
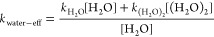
*k*_H_2_O_ and *k*_(H_2_O)_2__ are the bimolecular rate constants for the reaction of MACR oxide with water monomers and dimers, respectively. Lin et al. found that the value of *k*_water – eff_ is ≈80 times smaller than previous theoretical estimates,^62^ giving rise to the suggestion that anti-MACR oxide is surprisingly long lived in humid environments, at 298 K.^24^

We now use the estimated temperature dependence of the rate constants *k*_H_2_O_ and *k*_(H_2_O)_2__ provided by Lin et al. for both anti- and syn-MACR oxides to determine the contribution of bimolecular reactivity with water vapor at the different temperatures considered in our work. In Table 4, we show the rate constants *k*_H_2_O_ and *k*_(H_2_O)_2__ for anti-MACR oxide and syn-MACR oxide at temperatures of 288.8, 298.0, and 310.0 K. Also shown in Table 4 are the water and water dimer concentrations appropriate for two different relative humidities: 35 and 70%. These data are then combined to calculate *k*_water – eff_ and *k*_atm_, the rate constant that describes the total depletion of anti-MACR oxide,
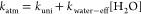


**Table 4:** Bimolecular and Total Depletion Rate Constants—Anti- and Syn-MACR Oxides^a^

temp (K)	rel. humid.	*k*_uni_ (s^–1^)	*k*_H_2_O_ (cm^3^ s^–1^)	*k*_(H_2_O)_2__ (cm^3^ s^–1^)	[H_2_O] (cm^–3^)	[(H_2_O)_2_] (cm^–3^)	*k*_water – eff_ (cm^3^ s^–1^)	*k*_atm_ (s^–1^)
Anti-MACR oxide								
288.8	35	4.0	4.2 × 10^–17^	2.3 × 10^–14^	1.6 × 10^17^	5.9 × 10^13^	5.0 × 10^–17^	12.
288.8	70	4.0	4.2 × 10^–17^	2.3 × 10^–14^	3.1 × 10^17^	2.4 × 10^14^	5.9 × 10^–17^	22.
298.0	35	9.7	4.9 × 10^–17^	1.6 × 10^–14^	2.7 × 10^17^	1.5 × 10^14^	5.8 × 10^–17^	25.
298.0	70	9.7	4.9 × 10^–17^	1.6 × 10^–14^	5.4 × 10^17^	5.9 × 10^14^	6.7 × 10^–17^	46.
310.0	35	29.	6.0 × 10^–17^	1.1 × 10^–14^	5.1 × 10^17^	4.4 × 10^14^	6.9 × 10^–17^	64.
310.0	70	29.	6.0 × 10^–17^	1.1 × 10^–14^	1.0 × 10^18^	1.8 × 10^15^	7.9 × 10^–17^	1.1 × 10^2^
Syn-MACR oxide								
288.8	35	2.0 × 10^3^	1.2 × 10^–20^	4.5 × 10^–17^	1.6 × 10^17^	5.9 × 10^13^	2.9 × 10^–20^	2.0 × 10^3^
288.8	70	2.0 × 10^3^	1.2 × 10^–20^	4.5 × 10^–17^	3.1 × 10^17^	2.4 × 10^14^	4.6 × 10^–20^	2.0 × 10^3^
298.0	35	3.7 × 10^3^	1.4 × 10^–20^	4.1 × 10^–17^	2.7 × 10^17^	1.5 × 10^14^	3.7 × 10^–20^	3.7 × 10^3^
298.0	70	3.7 × 10^3^	1.4 × 10^–20^	4.1 × 10^–17^	5.4 × 10^17^	5.9 × 10^14^	5.9 × 10^–20^	3.7 × 10^3^
310.0	35	8.1 × 10^3^	1.8 × 10^–20^	3.6 × 10^–17^	5.1 × 10^17^	4.4 × 10^14^	4.9 × 10^–20^	8.1 × 10^3^
310.0	70	8.1 × 10^3^	1.8 × 10^–20^	3.6 × 10^–17^	1.0 × 10^18^	1.8 × 10^15^	8.1 × 10^–20^	8.1 × 10^3^

a The buffer gas is N_2_ at a pressure of 1013 mbar in all cases.

The values for *k*_uni_ are taken from our master equation data (see Figures 3 and 4). The details of these calculations are presented in the Supporting Information, along with results from a full range of atmospheric conditions, 288–320 K (Table S3).

Several trends are evident in these data. First, and as discussed above, anti- and syn-MACR oxides undergo unimolecular decomposition at dramatically different rates, and at rates that are sharply dependent on atmospheric conditions. (Compare the values of *k*_uni_ in the top and bottom halves of Table 4.) Second, as reported by Anglada and Solé^62^ and Lin et al.,^24^ anti- and syn-MACR oxides differ significantly in their bimolecular reactivity toward water monomers and water dimers. (Compare the values of *k*_water – eff_ in top and bottom halves of Table 4.) Anti-MACR oxide, which is relatively stable with respect to unimolecular decay, reacts relatively fast with water monomers and water dimers. In contrast, the unimolecular decay of syn-MACR oxide is significantly faster than the anti-species, and the bimolecular reaction of the syn-species is ≈ 1000× slower than the anti-species. The result is that the loss of the syn-conformers is dominated by unimolecular decay, with bimolecular reactions with water playing a negligible role: for these molecules *k*_atm_= *k*_uni_. On the other hand, for the anti-conformers, the slower unimolecular decay, and faster bimolecular rate, means that bimolecular decay dominates the loss of anti-MACR oxide. The dominance of the bimolecular decay for the anti-conformers is not constant; however: *k*_atm_, the total decay rate constant is 5.5× that of the unimolecular decay constant at the average surface temperature of 288.8 K and a relative humidity of 70%, but this ratio drops to 2.2 if the temperature is 310 K and the relative humidity is 35%. Using data presented in Table S3, we see that these trends persist at the full range of atmospheric conditions considered. For the syn-conformers, *k*_atm_= *k*_uni_ always. For the anti-conformers, the unimolecular pathway takes on a more important role at in the total decay at higher temperatures. This is especially notable under lower humidity conditions.

The variable, and conformer dependent, lifetime of MACR oxide under atmospheric conditions has the potential to impact the oxidation of SO_2_, and thus the formation of atmospheric aerosols. The bimolecular rate constant for the reaction of SO_2_ with MACR oxide at 298 K has been measured to be 1.5 ± 0.4 × 10^–10^ cm^3^ s^–1^.^24^ While the temperature dependence of this rate constant has not been determined, the corresponding reaction of SO_2_ with MVK oxide is found to have a negative temperature dependence corresponding to a negative activation barrier of −3.7 ± 0.4 kcal/mol.^25^ If similar temperature dependent behavior is observed for the reaction of MACR oxide with SO_2_, then the second-order rate constant for this reaction will be ≈ 20% higher at the average atmospheric temperature for the surface (288.8 K) compared to 298 K and will be ≈ 35% lower at the highest temperature considered in our work (320 K), compared to 298 K.

The concentration of SO_2_ in the atmosphere is highly variable, and dependent on local conditions, volcanic activity, point sources of pollution, and weather conditions. Surface concentrations of 3–5 ppb are not unusual in urban environments, although they decrease nearly exponentially with increasing altitude, and are 1 ppt or less at 3 km.^61,69^ In isolated coal-fueled power plant plumes, concentrations can exceed 200 ppb, however.^61^

These approximate rate constants and order of magnitude estimates of SO_2_ concentrations allow us to gauge the impact of MACR oxide on the oxidation of SO_2_, a critical first step in the formation of sulfur-based atmospheric aerosols. For example, in urban environments, a SO_2_ concentration of 4 ppb and a bimolecular rate constant (288.8 K) of 1.8 × 10^–10^ cm^3^ s^–1^ provide a pseudo-first-order rate constant of 18 s^–1^. As summarized in Table 5, these approximate calculations suggest that the rate constant for the oxidation of SO_2_ is comparable to *k*_atm_ for anti-MACR oxide under these conditions: SO_2_ oxidation is competitive with our composite measure of the reaction of anti-MACR oxide with water vapor and unimolecular decay. In localized environments that are SO_2_ rich (≈ 200 ppb), but near the surface, such as power plant plumes, the SO_2_ oxidation pseudo-first-order rate constant (*k*_SO_2__) can be close to 900 s^–1^. Inspection of Table 5 shows that this value greatly exceeds the composite rate of reaction of anti-MACR oxide with water vapor and unimolecular decay and is a significant factor in the overall reactivity of syn-MACR oxide. These results are more apparent at lower temperatures where *k*_atm_ is smaller and *k*_SO_2__ is larger. While the lack of documented temperature dependence for the SO_2_ oxidation reaction rate constant renders these calculations semi-quantitative, at best, the conclusion is clear: anti-MACR oxide has a sufficiently long lifetime relative to unimolecular decay, and relative to bimolecular reactivity with water vapor, to serve as an important oxidant for SO_2_ under conditions commonly encountered at the surface. Importantly, in regions of localized high SO_2_ concentrations, even the rapidly decaying syn-MACR oxide conformers are found to have an atmospheric lifetime sufficient to serve as an SO_2_ oxidant.

**Table 5:** Extrapolated Pseudo First-Order Rate Constants (*k*_SO2_) for the Reaction of SO_2_ with MACR Oxide^a^

	*k*_SO2_ (s^–1^)		
	*T* = 288.8 K	*T* = 298.0 K	*T* = 310.0 K
urban environments; [SO_2_] ≈ 4 ppb^b^	18.	15.	12.
power plant plumes; [SO_2_] ≈ 200 ppb^c^	885.	738.	590.
			
*k*_atm_ (s^–1^)—syn-MACR oxide^d^	2.0 × 10^3^	3.7 × 10^3^	8.1 × 10^3^
			
*k*_atm_ (s^–1^)—anti-MACR oxide^d^	12.–22.	25.–46.	64.–1.1 × 10^2^

a Composite rate constants (*k*_atm_) for the rate of unimolecular decay and bimolecular reaction with water are also shown for comparison (see also Table 4).

b Ref (69).

c Ref (61).

d Values taken from Table 4 for ease of comparison. For the anti-conformer, the range of values represents relative humidities of 35 and 70%.

## V Conclusions

Methacrolein oxide (MACR oxide), one of the four-carbon Criegee intermediates formed from the ozonolysis of isoprene, is subject to rapid cis-trans isomerization under atmospheric conditions. The rate of cis-trans conformational change is strongly dependent on atmospheric conditions. No evidence for conversion between the syn- and anti-conformers of MACR oxide is observed on timescales competitive with other unimolecular or bimolecular processes under atmospheric conditions.

Similarly, the rates of unimolecular decay of syn- and anti-MACR oxide to form dioxole and dioxirane structures, respectively, are dependent on atmospheric conditions. Specifically, these rates increase with increasing temperature within the troposphere. A comparison of the temperature-dependent unimolecular decay rates with the also temperature-dependent bimolecular rates of reactions of MACR oxide with water vapor reveals that, while anti-MACR oxide is lost primarily by reaction with water at all reasonable temperature and humidity conditions in the troposphere, the unimolecular decay is more important for this species at higher temperature. This is naturally a more important trend under low-humidity conditions. For syn-MACR oxide, unimolecular decay dominates under all conditions. The enhanced lifetime of anti-MACR oxide, coupled with SO_2_ concentrations that are also stratified by atmospheric conditions, suggests that isoprene-derived Criegee intermediates can play an important role in the formation of sulfate-based atmospheric aerosols.

## Supporting Information Available

Additional tabulated data and figures, details on calculations, tabulation of hindered rotor potentials, and tabulation of optimized structures (PDF)
