# Supplementary material for: Master Equation Studies of the Unimolecular Decay of Thermalized Methacrolein Oxide: The Impact of Atmospheric Conditions
Source: J Phys Chem A. 2023 May 10;127(20):4492–502. doi: 10.1021/acs.jpca.3c00542 (PMC10226126; doi:10.1021/acs.jpca.3c00542)
Supplement: Supplementary file 1 — jp3c00542_si_001.pdf [file jp3c00542_si_001.pdf]

Supporting Information

Master Equation Studies of the Unimolecular Decay of Thermalized Methacrolein Oxide: Impact of Atmospheric Conditions

Hyun Kyung Lee [a], Pitchaya Chantanapongvanij [a], Rory R. Schmidt [a], and Thomas A. Stephenson [a]*

[a] Department of Chemistry and Biochemistry, Swarthmore College, 500 College Avenue, Swarthmore, PA 19081 USA

* Email: tstephe1@swarthmore.edu

Contents

Page

Table S1: Methacrolein oxide cis-trans conformer equilibration rates and transient population ratios. S3

Figure S1: Initial population evolution of anti-cis and anti-trans conformers S3

Table S2: Methacrolein oxide conformer unimolecular decay rate constants. S4

Calculation Details: Reactivity of MACR oxide with water monomers and water dimers: temperature dependence. S4

Table S3: Bimolecular and total depletion rate constants – anti- and syn-MACR oxide S5

Figure S2: Pressure dependence of methacrolein oxide unimolecular decay rate constants at 298.0 K. S6

Plots of hindered rotor potentials used in master equation calculations. S7-S11

Optimized structures of all species used in master equation simulations; B2PLYPD3/cc-PVTZ level of theory. S12-S17

Table S1: Methacrolein oxide cis-trans conformer equilibration rates and transient population ratios. The buffer gas is N_2 in all cases.

Temp. (K)	Pressure (mbar)	Syn equilibration rate cst (s^{-1})	Syn population ratio (cis:trans)	Anti equilibration rate cst (s^{-1})	Anti population ratio (trans:cis)
259.3	542.0	8.0×10^6	21.4	1.4×10^7	408.
288.8	1013.0	2.6×10^7	15.8	4.3×10^7	221.
295.0	1013.0	3.0×10^7	14.9	4.9×10^7	197.
298.0	1013.0	3.3×10^7	14.4	5.2×10^7	187.
305.0	1013.0	3.8×10^7	13.6	5.9×10^7	166.
310.0	1013.0	4.3×10^7	13.0	6.6×10^7	145.
315.0	1013.0	4.7×10^7	12.5	7.2×10^7	141.
320.0	1013.0	5.2×10^7	12.0	7.8×10^7	131.

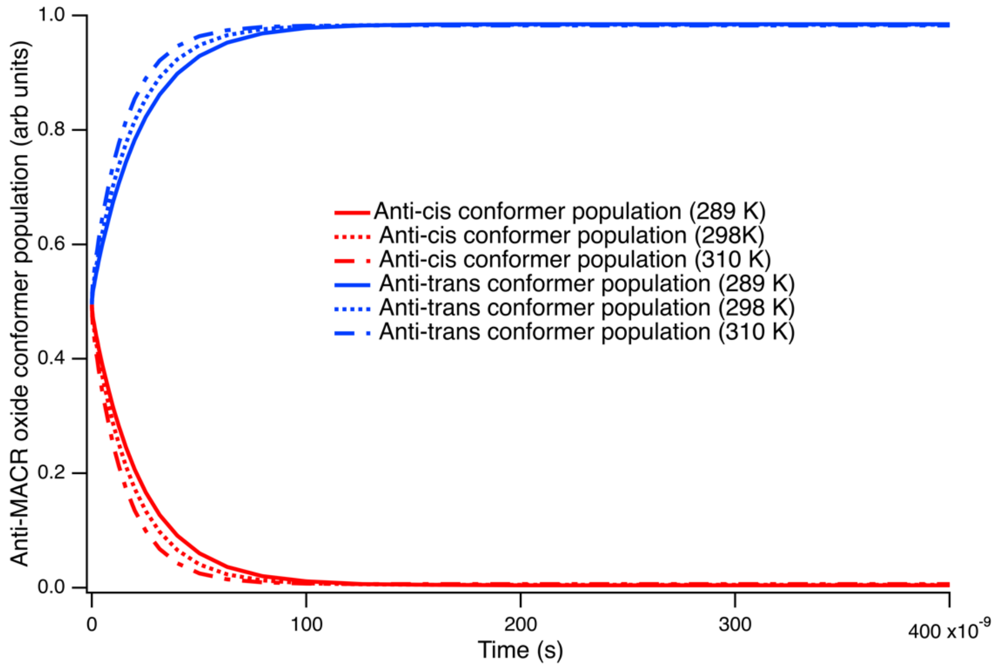

Figure S1: Initial population evolution of anti-cis (red) and anti-trans (blue) conformers at temperatures of 288.8 K (solid lines), 298 K (dashed lines), and 310 K (dot-dash lines). The buffer gas is N_2 at a pressure of 1013 mbar in all cases.

Table S2: Methacrolein oxide conformer unimolecular decay rate constants. The buffer gas is N_2 in all cases.

Temp. (K)	Pressure (mbar)	Syn decay rate cst (s^{-1})	Anti decay rate cst (s^{-1})	Dioxirane population ratio (trans:cis)
259.3	542.0	1.8×10^2	0.14	4.27
288.8	1013.0	2.0×10^3	3.9	3.63
295.0	1013.0	3.1×10^3	7.3	3.53
298.0	1013.0	3.7×10^3	9.7	3.49
305.0	1013.0	5.9×10^3	18.	3.38
310.0	1013.0	8.1×10^3	29.	3.32
315.0	1013.0	1.1×10^4	44.	3.26
320.0	1013.0	1.5×10^4	67.	3.20

Calculation Details: Reactivity of MACR oxide with water monomers and water dimers: temperature dependence.

To extract the temperature dependence of the rate constants k_{H_2O} and $k_{(H_2O)_2}$, we have adopted the data of Lin, et al., (see Supplemental Table 5 in *Comm. Chem*. **2021**, *4*, 1) and made fits to Arrhenius expressions. The results are:

Anti-MACR oxide + H_2O: $ln\mathrm{k} = -32.448 - 1521/T$

Anti-MACR oxide + $(H_2O)_2$: $ln\mathrm{k} = -41.908 + 3027.8/T$

Syn-MACR oxide + H_2O: $ln\mathrm{k} = -40.292 - 1608.6/T$

Syn-MACR oxide +$(H_2O)_2$: $ln\mathrm{k} = -40.594 + 850.38/T$

These expressions, along with ambient temperatures in the atmosphere, are used to calculate the values of k_{H_2O} and $k_{(H_2O)_2}$ that appear in Tables 4 and S3.

Water vapor concentrations as a function of temperature are taken from *Lange's Handbook of Chemistry*, 17th edition, (McGraw-Hill, New York, 2017), pp 240 -241.

Water dimer concentrations are calculated using temperature dependent equilibrium constants taken from B. Ruscic, *J. Phys. Chem. A* **2013**, *117*, 10381 (Table S4).

Table S3: Bimolecular and total depletion rate constants – anti- and syn-MACR oxide. The buffer gas is N_2 at a pressure of 1013 mbar in all cases.

Temp (K)	Rel. Humid.	k_{uni} (s^{-1})	k_{H_2O} ($cm^3\ s^{-1}$)	$k_{(H_2O)_2}$ ($cm^3\ s^{-1}$)	[H_2O] (cm^{-3})	[$(H_2O)_2$] (cm^{-3})	$k_{water-eff}$ ($cm^3\ s^{-1}$)	k_{atm} (s^{-1})
**Anti-MACR oxide**								
288.8	35	4.0	4.2×10^{-17}	2.3×10^{-14}	1.6×10^{17}	5.9×10^{13}	5.0×10^{-17}	12.
288.8	70	4.0	4.2×10^{-17}	2.3×10^{-14}	3.1×10^{17}	2.4×10^{14}	5.9×10^{-17}	22.
295.0	35	7.3	4.7×10^{-17}	1.8×10^{-14}	2.3×10^{17}	1.1×10^{14}	5.6×10^{-17}	20.
295.0	70	7.3	4.7×10^{-17}	1.8×10^{-14}	4.6×10^{17}	4.6×10^{14}	6.5×10^{-17}	37.
298.0	35	9.7	4.9×10^{-17}	1.6×10^{-14}	2.7×10^{17}	1.5×10^{14}	5.8×10^{-17}	25.
298.0	70	9.7	4.9×10^{-17}	1.6×10^{-14}	5.4×10^{17}	5.9×10^{14}	6.7×10^{-17}	46.
305.0	35	18.	5.5×10^{-17}	1.3×10^{-14}	3.9×10^{17}	2.8×10^{14}	6.4×10^{-17}	44.
305.0	70	18.	5.5×10^{-17}	1.3×10^{-14}	7.8×10^{17}	1.1×10^{15}	7.4×10^{-17}	76.
310.0	35	29.	6.0×10^{-17}	1.1×10^{-14}	5.1×10^{17}	4.4×10^{14}	6.9×10^{-17}	64.
310.0	70	29.	6.0×10^{-17}	1.1×10^{-14}	1.0×10^{18}	1.8×10^{15}	7.9×10^{-17}	1.1×10^{2}
315.0	35	44.	6.5×10^{-17}	9.4×10^{-15}	6.6×10^{17}	6.7×10^{14}	7.4×10^{-17}	93.
315.0	70	44.	6.5×10^{-17}	9.4×10^{-15}	1.3×10^{18}	2.7×10^{15}	8.4×10^{-17}	1.5×10^{2}
320.0	35	67.	7.0×10^{-17}	8.1×10^{-15}	8.4×10^{17}	1.0×10^{15}	8.0×10^{-17}	1.4×10^{2}
320.0	70	67.	7.0×10^{-17}	8.1×10^{-15}	1.7×10^{18}	4.0×10^{15}	8.9×10^{-17}	2.2×10^{2}
**Syn-MACR oxide**								
288.8	35	2.0×10^{3}	1.2×10^{-20}	4.5×10^{-17}	1.6×10^{17}	5.9×10^{13}	2.9×10^{-20}	2.0×10^{3}
288.8	70	2.0×10^{3}	1.2×10^{-20}	4.5×10^{-17}	3.1×10^{17}	2.4×10^{14}	4.6×10^{-20}	2.0×10^{3}
295.0	35	3.1×10^{3}	1.4×10^{-20}	4.2×10^{-17}	2.3×10^{17}	1.1×10^{14}	3.4×10^{-20}	3.1×10^{3}
295.0	70	3.1×10^{3}	1.4×10^{-20}	4.2×10^{-17}	4.6×10^{17}	4.6×10^{14}	5.6×10^{-20}	3.1×10^{3}
298.0	35	3.7×10^{3}	1.4×10^{-20}	4.1×10^{-17}	2.7×10^{17}	1.5×10^{14}	3.7×10^{-20}	3.7×10^{3}
298.0	70	3.7×10^{3}	1.4×10^{-20}	4.1×10^{-17}	5.4×10^{17}	5.9×10^{14}	5.9×10^{-20}	3.7×10^{3}
305.0	35	5.9×10^{3}	1.6×10^{-20}	3.8×10^{-17}	3.9×10^{17}	2.8×10^{14}	4.4×10^{-20}	5.9×10^{3}
305.0	70	5.9×10^{3}	1.6×10^{-20}	3.8×10^{-17}	7.8×10^{17}	1.1×10^{15}	7.1×10^{-20}	5.9×10^{3}
310.0	35	8.1×10^{3}	1.8×10^{-20}	3.6×10^{-17}	5.1×10^{17}	4.4×10^{14}	4.9×10^{-20}	8.1×10^{3}
310.0	70	8.1×10^{3}	1.8×10^{-20}	3.6×10^{-17}	1.0×10^{18}	1.8×10^{15}	8.1×10^{-20}	8.1×10^{3}
315.0	35	1.1×10^{4}	1.9×10^{-20}	3.5×10^{-17}	6.6×10^{17}	6.7×10^{14}	5.5×10^{-20}	1.1×10^{4}
315.0	70	1.1×10^{4}	1.9×10^{-20}	3.5×10^{-17}	1.3×10^{18}	2.7×10^{15}	9.1×10^{-20}	1.1×10^{4}
320.0	35	1.5×10^{4}	2.1×10^{-20}	3.3×10^{-17}	8.4×10^{17}	1.0×10^{15}	6.1×10^{-20}	1.5×10^{4}
320.0	70	1.5×10^{4}	2.1×10^{-20}	3.3×10^{-17}	1.7×10^{18}	4.0×10^{15}	1.0×10^{-19}	1.5×10^{4}

Figure S2: Pressure dependence of methacrolein oxide unimolecular decay rate constants at 298.0 K.   Upper panel: syn-MACR oxide to dioxole. Middle panel: anti-MACR oxide to dioxirane. Lower panel: syn-MACR oxide to dioxirane.

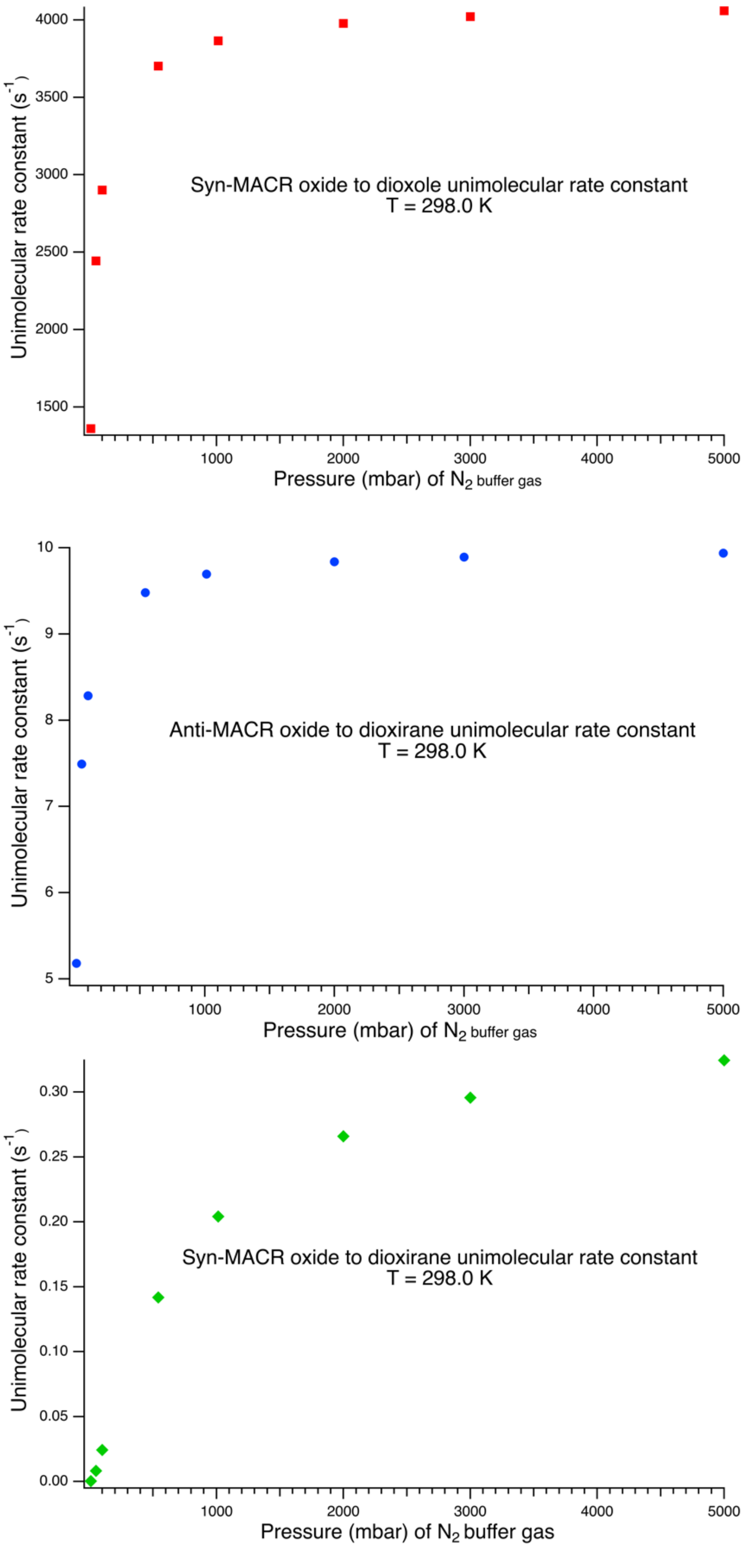

Plots of hindered rotor potentials used in master equation calculations.

Methyl rotor potentials

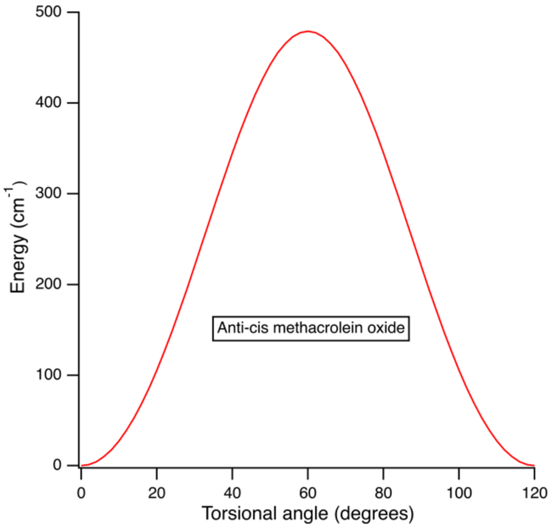

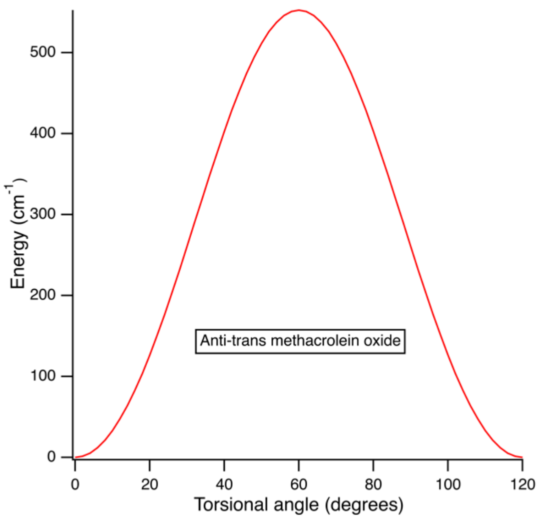

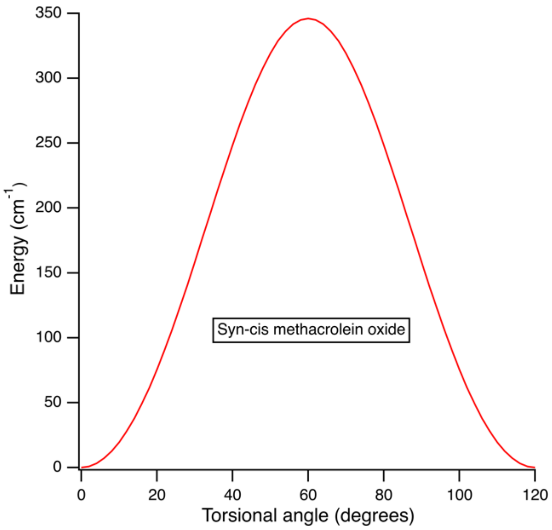

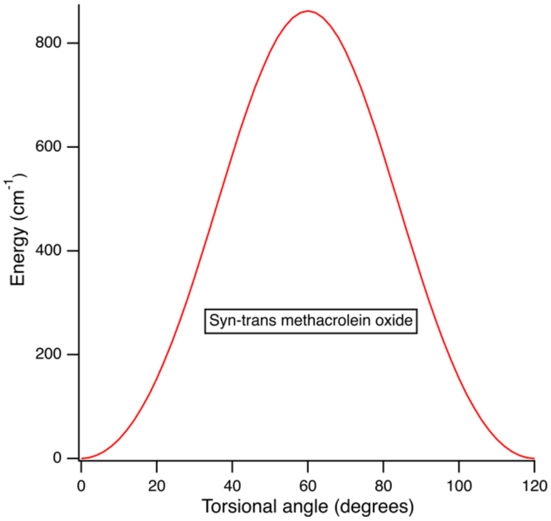

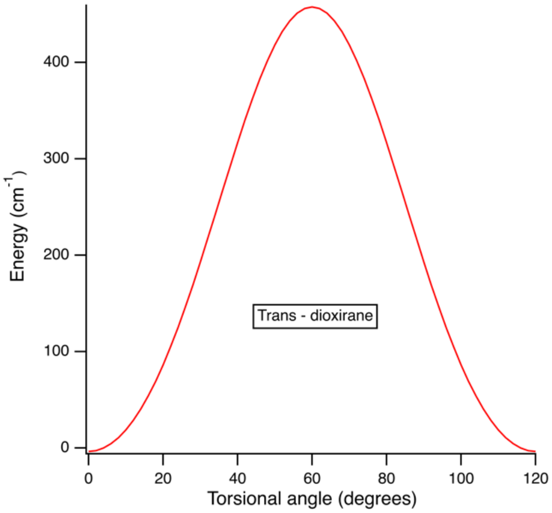

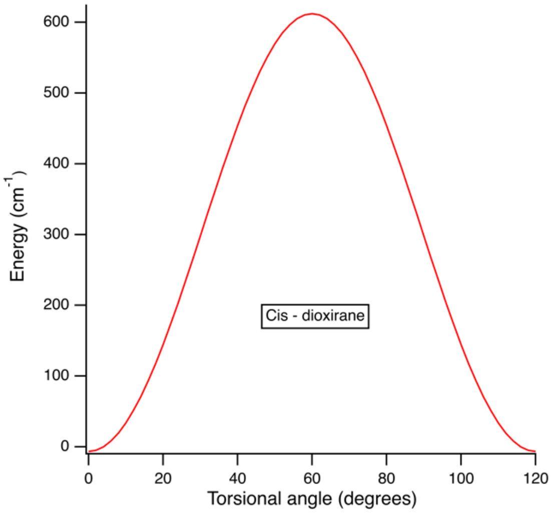

Methyl rotor potentials (continued)

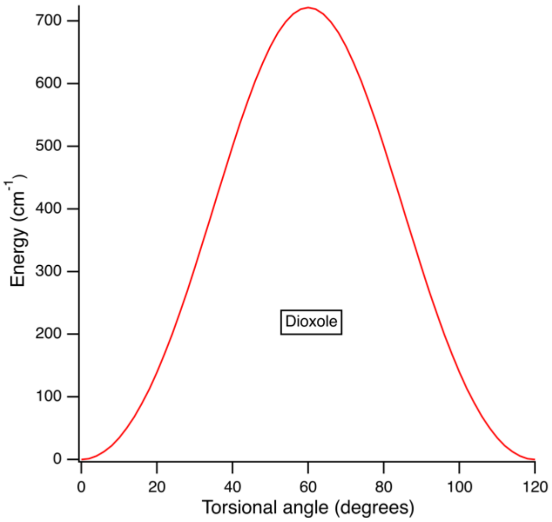

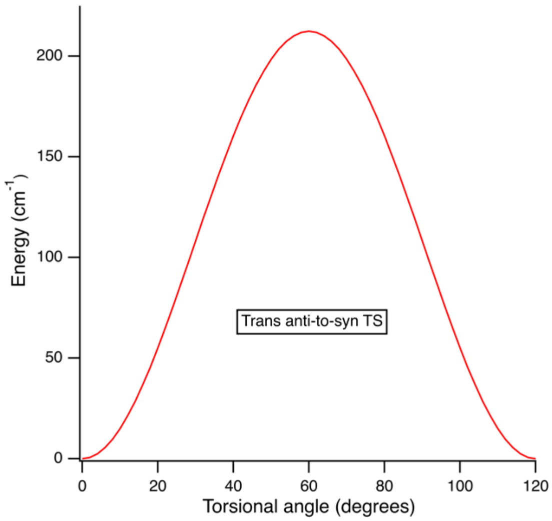

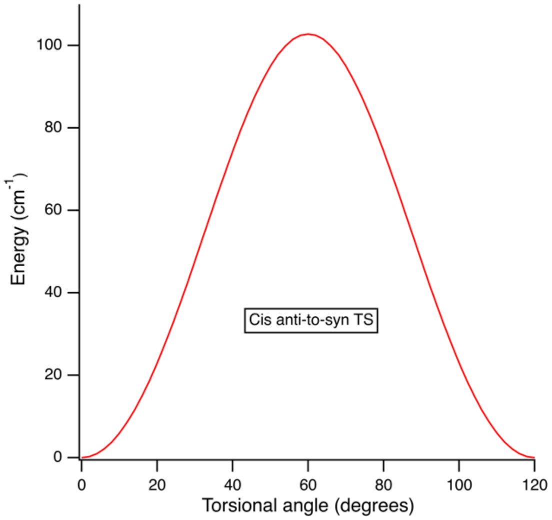

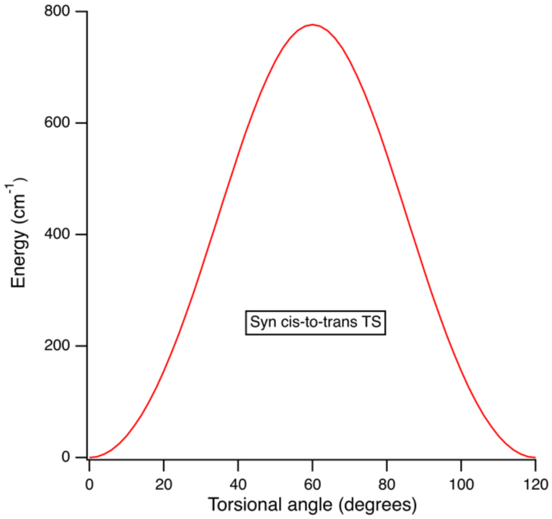

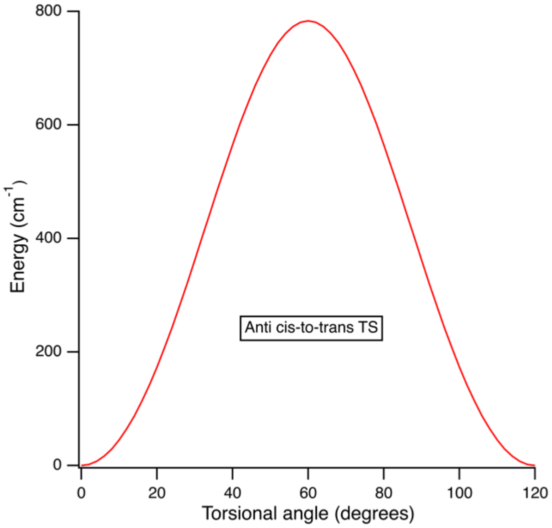

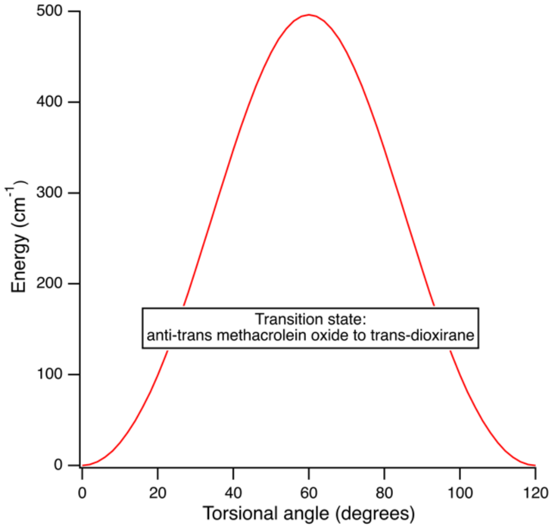

Methyl rotor potentials (continued)

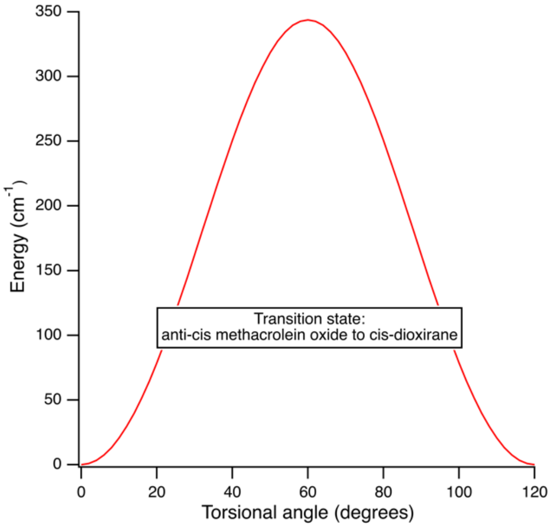

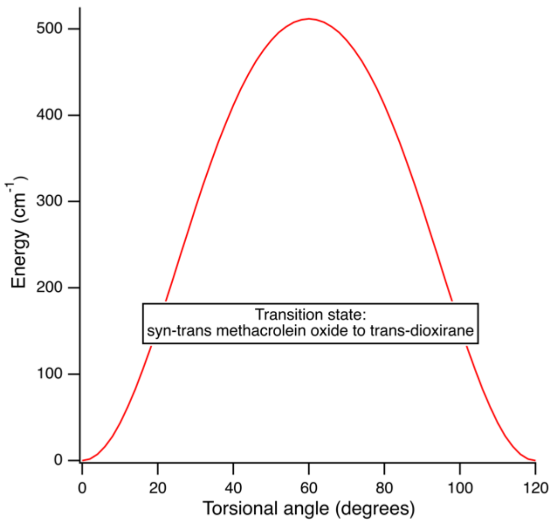

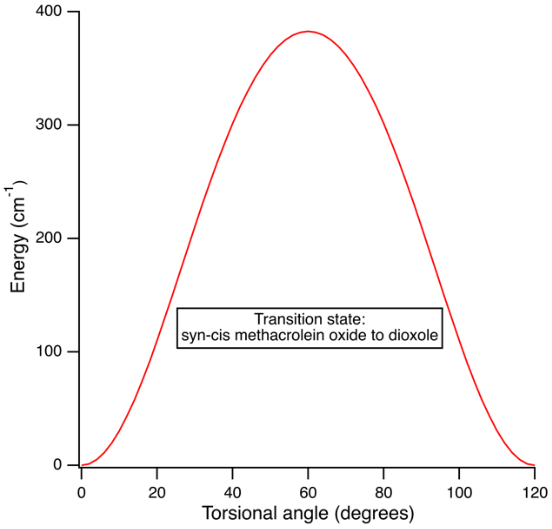

<u>Carbon-carbon bond torsional potentials</u>

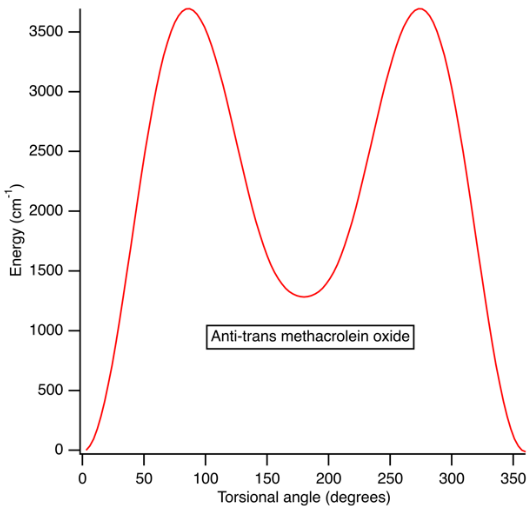

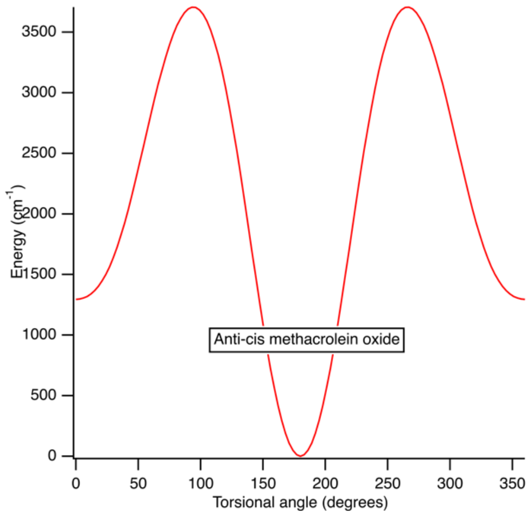

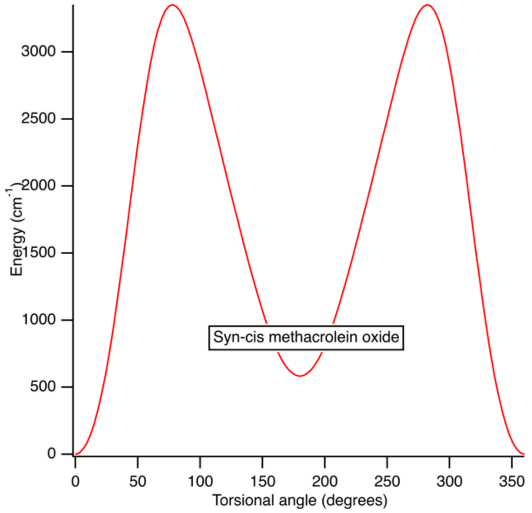

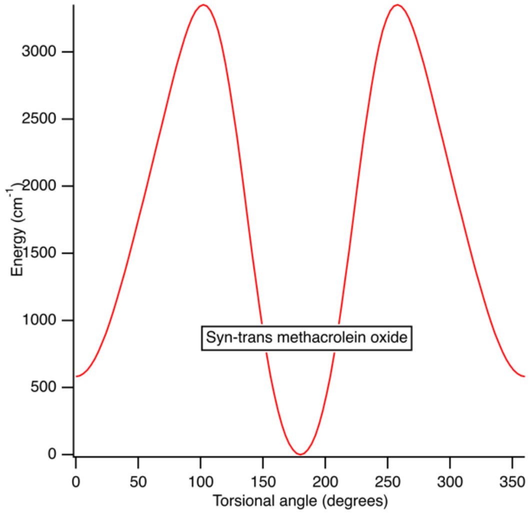

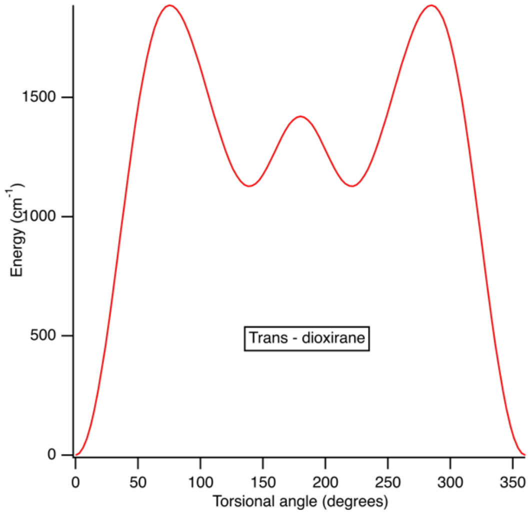

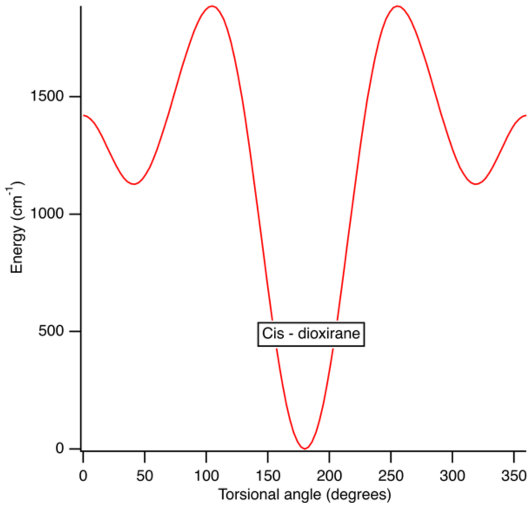

Carbon-carbon bond torsional potentials (continued)

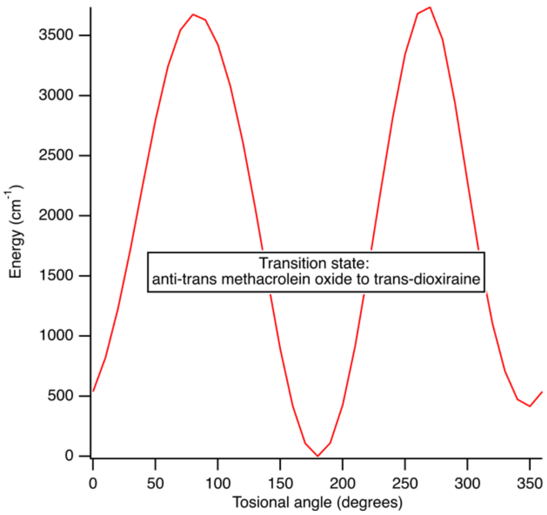

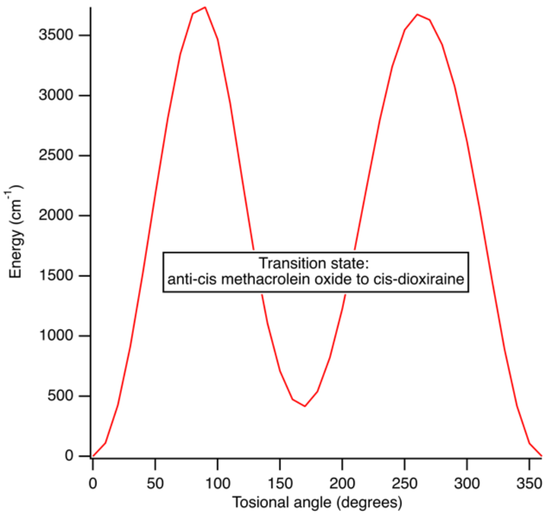

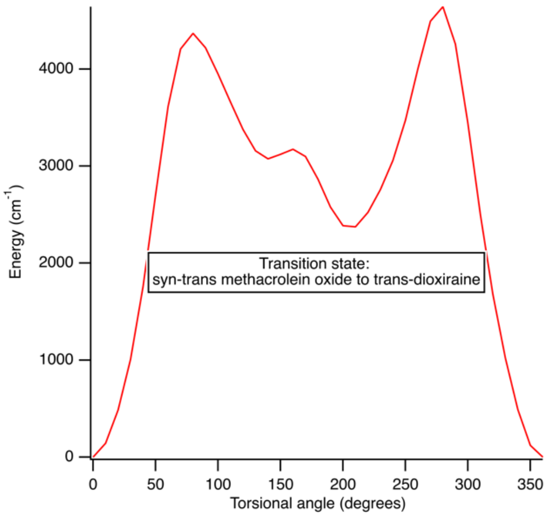

Optimized structures of all species used in master equation simulations; B2PLYPD3/cc-PVTZ level of theory.

Anti-trans methacrolein oxide

C	0.99165400	-0.11168900	0.00000000
C	1.96593900	-1.04016900	-0.00000100
H	3.00759600	-0.75714000	-0.00000100
H	1.73529400	-2.09587100	-0.00000100
C	-0.36733500	-0.56498400	0.00000000
H	-0.66444000	-1.60653600	0.00000000
O	-1.31061800	0.30054900	0.00000000
O	-2.57944600	-0.13393900	0.00000100
C	1.25768800	1.36547300	0.00000000
H	0.81384300	1.83800000	-0.87509500
H	0.81384700	1.83799900	0.87509700
H	2.32670200	1.55888200	-0.00000300

Anti-cis methacrolein oxide

C	0.96068600	0.09260700	-0.00000200
C	1.18917600	1.41608700	-0.00000400
H	2.19827700	1.79899100	0.00000200
H	0.37882500	2.12911500	-0.00001100
C	-0.37327700	-0.46088400	-0.00000800
H	-0.57931500	-1.52458100	-0.00002900
O	-1.39821100	0.30489800	0.00001100
O	-2.61685700	-0.24945800	-0.00000200
C	2.06813100	-0.92620500	0.00000400
H	2.00792100	-1.56933700	0.87829700
H	2.00792900	-1.56934000	-0.87828500
H	3.03861700	-0.43799400	0.00000900

Syn-trans methacrolein oxide

C	-0.87256700	-0.01850000	-0.00000500
C	-2.09754500	-0.58265300	0.00001300
H	-2.98994900	0.02493600	0.00001500
H	-2.22768100	-1.65553400	0.00001600
C	0.22561800	-0.94973300	-0.00001100
H	0.03251200	-2.01380000	-0.00001800
O	1.48565200	-0.71608500	-0.00000400

O	1.96686300	0.53898700	0.00001500
C	-0.68190300	1.47154100	-0.00000900
H	-0.11131900	1.78818900	0.86827100
H	-0.11130400	1.78818500	-0.86827800
H	-1.65400400	1.96088700	-0.00001500

Syn-cis methacrolein oxide

C	0.79416700	0.12390000	0.00001400
C	0.54288100	1.45147300	0.00000300
H	1.37460300	2.14319300	0.00002500
H	-0.46460900	1.82340600	-0.00001000
C	-0.22555000	-0.87808500	0.00000100
H	0.03101800	-1.92865400	0.00002400
O	-1.50592800	-0.73549600	0.00000400
O	-2.05878100	0.48587400	-0.00000900
C	2.20194600	-0.42233000	-0.00000900
H	2.38592600	-1.04002900	0.87906000
H	2.38588300	-1.04006400	-0.87906500
H	2.92418000	0.38938100	-0.00004400

Trans-dioxirane

C	-0.80328200	-0.08119600	0.00000000
C	-1.82119800	-0.94225300	0.00000000
H	-2.84729900	-0.60510100	0.00000000
H	-1.65186400	-2.01008100	-0.00000100
C	-0.94903100	1.40904100	0.00000000
H	-0.46386500	1.83945800	0.87469300
H	-0.46388000	1.83945700	-0.87470200
H	-1.99781500	1.69371300	0.00000800
C	0.57232900	-0.63088500	-0.00000100
H	0.65062700	-1.71517300	-0.00000100
O	1.54882300	0.02684300	0.75386800
O	1.54882500	0.02684300	-0.75386700

Cis-dioxirane

C	0.75509700	0.15491800	0.06310600
C	0.93030600	1.45453100	-0.16011900
H	1.90634600	1.85230100	-0.39748100

H	0.09916800	2.14115100	-0.11434600
C	1.84648500	-0.87281700	0.01639100
H	1.93964700	-1.39156000	0.97211800
H	1.63426400	-1.63029600	-0.73991600
H	2.80357500	-0.41466800	-0.21886700
C	-0.59388800	-0.37413300	0.39814300
H	-0.62685800	-1.09523200	1.21383200
O	-1.47798100	-0.66940600	-0.65050900
O	-1.69503700	0.46481900	0.32295100

Dioxole

C	0.16060400	1.16190200	0.03274200
C	-0.70781800	-0.06193100	-0.00426300
C	0.11443700	-1.10237600	0.00157500
H	0.03607200	1.73261300	0.96042700
H	-0.00480200	1.82731000	-0.81987800
H	-0.09202200	-2.16033800	0.00135000
O	1.44196300	-0.81409700	0.02042600
O	1.50672500	0.66590400	-0.04726400
C	-2.19440800	-0.02680300	-0.01148900
H	-2.61006200	-1.03192800	-0.01901900
H	-2.58147700	0.49125700	0.86817000
H	-2.57410500	0.50187800	-0.88774100

Methacrolein oxide: anti trans-to-cis transition state

O	-2.60732400	-0.06242400	-0.03872700
O	-1.31508100	-0.01510800	-0.39606100
C	-0.42530400	0.10111900	0.50454000
C	0.99100400	0.11844200	0.09181500
C	1.60944600	1.28090700	-0.10772800
H	2.65108400	1.31244600	-0.39441100
H	1.09445500	2.22316900	0.00586600
H	-0.77850600	0.14134200	1.52968500
C	1.65288500	-1.22412400	-0.05377300
H	1.15465800	-1.81026000	-0.82633700
H	2.70117900	-1.11045000	-0.31961200
H	1.58819000	-1.79404800	0.87399300

Methacrolein oxide: syn trans-to-cis transition state

C	-0.41636400	-0.33153900	0.84690700
O	-1.58039400	-0.28821600	0.32385000
O	-1.74539300	0.20646700	-0.90952700
C	0.80936300	0.11637200	0.17303400
C	1.09872600	1.41311000	0.10359900
H	2.00786300	1.75173300	-0.37231100
H	0.43282700	2.16331800	0.50237500
C	1.66738100	-0.97895700	-0.39625200
H	2.57750500	-0.56664400	-0.82545200
H	1.94109800	-1.70826700	0.36734100
H	1.12153600	-1.50939100	-1.17697600
H	-0.42917100	-0.79067300	1.82670700

Methacrolein oxide: trans anti-to-syn transition state

C	-0.90273700	-0.06841900	0.02912500
C	-2.10070400	-0.70449700	-0.21941700
H	-3.00839700	-0.14120600	-0.36508600
H	-2.16029200	-1.78110700	-0.27461600
C	0.23867800	-0.82170700	0.21655400
H	0.29513200	-1.89772800	0.17092000
O	1.45509000	-0.19649800	0.52032100
O	2.13563300	0.11205500	-0.58733200
C	-0.81632400	1.43478800	0.08969600
H	-0.34170700	1.76221500	1.01266000
H	-0.21654900	1.81686400	-0.73451200
H	-1.80744600	1.87551700	0.03097100

Methacrolein oxide: cis anti-to-syn transition state

C	0.82730100	0.07223300	-0.05672100
C	0.69738900	1.44864000	-0.13611600
H	1.55932400	2.08578900	-0.01552200
H	-0.25751400	1.91358000	-0.31645200
C	-0.24209200	-0.77582400	-0.20842300
H	-0.19627400	-1.85213400	-0.16494600
O	-1.52171300	-0.28363800	-0.50070500
O	-2.15861500	0.12916300	0.59752900
C	2.17709400	-0.54690000	0.21289400
H	2.13762000	-1.63233900	0.15958700

H	2.91019300	-0.19762000	-0.51188600
H	2.53112800	-0.27036300	1.20481900

Anti-trans methacrolein oxide to trans-dioxirane transition state

C	-0.92669600	-0.11451700	-0.02854200
C	0.44916800	-0.46265700	-0.28098100
O	1.34294000	0.45632700	-0.41314800
O	2.28504600	-0.24221100	0.46096500
H	0.75476000	-1.49180800	-0.41870500
C	-1.28068700	1.33382800	0.12320800
H	-1.00140800	1.89388100	-0.76806400
H	-2.34666800	1.45614900	0.29462400
H	-0.73201000	1.77007700	0.95649300
C	-1.80331100	-1.12862800	0.07228800
H	-1.47866200	-2.15422600	-0.03327600
H	-2.85074500	-0.95516100	0.27055400

Anti-cis methacrolein oxide to cis-dioxirane transition state

C	-0.89433400	0.09294100	0.00105600
C	0.41632000	-0.47853600	0.18062000
O	1.41522400	0.26687100	0.52544300
O	2.30369800	-0.29723400	-0.46809500
H	0.60241800	-1.54036200	0.08766800
C	-2.06095500	-0.85223200	-0.04253500
H	-2.15456100	-1.40410600	0.89231000
H	-2.98938400	-0.31598700	-0.21890200
H	-1.93195200	-1.58372000	-0.84044900
C	-0.99604700	1.42707600	-0.13581100
H	-0.11023200	2.04325100	-0.15996100
H	-1.95756900	1.90833700	-0.23943000

Syn-trans methacrolein oxide to trans-dioxirane transition state

C	0.79702200	-0.05894400	-0.03071300
C	-0.38404100	-0.86505300	-0.17853500
O	-1.57334700	-0.44972800	-0.46758700
O	-1.61700200	0.45441700	0.68093700
H	-0.25228500	-1.94814600	-0.18988700
C	0.77289800	1.42323700	-0.25281800

H	0.45705000	1.93579000	0.65060400
H	1.76568800	1.76506600	-0.53977100
H	0.06104700	1.68579000	-1.03118600
C	1.92947100	-0.73839500	0.24140100
H	1.91867000	-1.80708500	0.40358500
H	2.88052100	-0.23400100	0.32384700

Syn-cis methacrolein oxide to dioxole transition state

C	0.18115200	-0.98839300	-0.18616600
C	-0.71634200	0.05534200	-0.15655800
O	1.44201800	-0.79104800	0.13327300
O	1.77782000	0.54085800	0.07165100
C	-0.22913200	1.36487500	-0.04999300
H	-0.81766400	2.04772800	0.56330600
H	0.41772700	1.83120900	-0.76297100
H	-0.08090700	-2.03611600	-0.19361400
C	-2.17296000	-0.24772200	0.09882100
H	-2.34171600	-1.30647400	0.28255700
H	-2.51552400	0.30498300	0.97499300
H	-2.79693000	0.05557200	-0.74028500
